# On the origins of arrestin and rhodopsin

**DOI:** 10.1186/1471-2148-8-222

**Published:** 2008-07-29

**Authors:** Carlos E Alvarez

**Affiliations:** 1 Center for Molecular and Human Genetics, The Research Institute at Nationwide Children's Hospital, Columbus, OH, 43205, USA; 2 Department of Pediatrics, The Ohio State University College of Medicine, Columbus, OH, 43210, USA; 3 Novartis Institutes of BioMedical Research, CH-4002 Basel, Switzerland

## Abstract

**Background:**

G protein coupled receptors (GPCRs) are the most numerous proteins in mammalian genomes, and the most common targets of clinical drugs. However, their evolution remains enigmatic. GPCRs are intimately associated with trimeric G proteins, G protein receptor kinases, and arrestins. We conducted phylogenetic studies to reconstruct the history of arrestins. Those findings, in turn, led us to investigate the origin of the photosensory GPCR rhodopsin.

**Results:**

We found that the arrestin clan is comprised of the Spo0M protein family in archaea and bacteria, and the arrestin and Vps26 families in eukaryotes. The previously known animal arrestins are members of the visual/beta subfamily, which branched from the founding "alpha" arrestins relatively recently. Curiously, we identified both the oldest visual/beta arrestin and opsin genes in Cnidaria (but not in sponges). The arrestin clan has 14 human members: 6 alphas, 4 visual/betas, and 4 Vps26 genes. Others recently showed that the 3D structure of mammalian Vps26 and the biochemical function of the yeast alpha arrestin PalF are similar to those of beta arrestins. We note that only alpha arrestins have PY motifs (known to bind WW domains) in their C-terminal tails, and only visual/betas have helix I in the Arrestin N domain.

**Conclusion:**

We identified ciliary opsins in Cnidaria and propose this subfamily is ancestral to all previously known animal opsins. That finding is consistent with Darwin's theory that eyes evolved once, and lends some support to Parker's hypothesis that vision triggered the Cambrian explosion of life forms. Our arrestin findings have implications on the evolution of GPCR signaling, and on the biological roles of human alpha arrestins.

## Background

G protein coupled receptors (GPCRs) are arguably the most important proteins in human evolution and medicine [1]. The rhodopsin class of GPCRs alone is the most highly represented protein family in mammals [2]. Humans have on the order of 799 [3] to 1,282 [4] GPCRs, and they are the protein family most commonly targeted by clinical drugs. GPCRs are transmembrane receptors that mediate the majority of extracellular signaling in mammals. They can sense diverse types of signals, including hormones, lipids, olfactants, tastants, ions, light, and soluble and surface-anchored peptides. Despite the name, not all GPCRs signal through G proteins. And many or most GPCRs also signal through non-G protein-mediated pathways. The hallmark of GPCRs is that they have seven transmembrane helices. Thus, the terms seven transmembrane receptor (7TMR) and GPCR are used interchangeably by some.

It has been extremely challenging to determine the evolution of the 7TMR superfamily due to the large number of 7TMRs and the existence of highly divergent subfamilies. However, this was initiated for fully sequenced genomes from several bilateral animals, two fungi, two plants and one alveolate [5]. Recently-sequenced genomes (e.g., from protists and basal metazoans) will soon begin to fill in key gaps. Notably, it is widely believed that 7TMRs do not represent a single superfamily [1,6]. That is based on the perceived weakness, or lack of, protein sequence similarity between many 7TMRs – most importantly between the major 7TMR families of archaea, bacteria and eukaryotes. But others claimed that analysis with PSI-BLAST and hidden Markov models suggest that bacterial, archaeal, and eukaryotic/animal 7TMRs are distantly related [7]. This issue generates much attention (and confusion) with regards to the evolution of vision [8-10]. Importantly, protein sequence analysis strongly suggests that bacteriorhodopsins of archaea/bacteria and rhodopsins of animals are separate 7TMR subfamilies that arose independently [6]. The 7TMR subfamily named after rhodopsin contains vast numbers of non-opsin genes, and there is no indication that opsins are among the earliest members. To our knowledge, the oldest known member of the Rhodopsin subfamily is from yeast [5].

The three intimate associates of GPCRs are heterotrimeric G proteins, G protein coupled receptor kinases (GRKs) and arrestins. Study of those proteins could offer clues about 7TMR evolution. Phylogenetic analysis of G alpha subunits is possible due to the presence of a signature protein sequence. In the Pfam database, G alphas can be seen in protists, plants and unikonts. GRKs have been reported from mammals down to nematode worms and insects [11]. However, their relationship to the large family of protein kinases may complicate the determination of the phylogeny of GRKs; we found no reported attempt to do this.

The main focus of this work is reconstructing the arrestin phylogeny. Arrestins regulate the inactivation [12-14], internalization [15,16], trafficking [17,18] and signaling [19,20] of transmembrane receptors of the 7TM and kinase classes (Reviewed by [21]). They are defined by the presence of two homologous protein sequences known as Arrestin N and C domains. Arrestins selectively bind receptors that are both in an activated conformation and phosphorylated [22]. This is possible because they have an autoinhibited resting state maintained by interacting polar and solvent-excluded residues in abutting arrestin-N and C domains [23,24]. Phosphates on activated receptors destabilize that "polar core" and allow binding of specific receptor residues at the fulcrum of, and on the concave faces of, the two arrestin domains. A second feature maintaining the basal conformation is the "three-element interaction" of hydrophobic residues in beta strand I, alpha helix I, and beta strand XX in the C-terminal tail [25]. Release of this interaction in activated arrestin thus frees the C-terminal tail, allowing it to bind clathrin and its adaptor AP-2. Arrestin activities vary according to their own modifications by phosphorylation and ubiquitination, their interactions with dozens of other proteins [26], and the specifics of their interactions with receptors (e.g., low or high receptor-arrestin affinity can determine whether receptors are recycled or degraded [17]).

Until recently, when fungal arrestins were discovered [27,28], it was believed that arrestins emerged in animals. Fruit flies and mosquitos each have two known visual arrestins and two beta arrestins [29]. The worm *C. elegans*, which lacks vision, has one known beta arrestin [30]. Humans are presumed to have four arrestins: two photoreceptor-specific visual arrestins and two nearly ubiquitous beta arrestins [26]. The first phylogenetic study of visual and beta arrestins was recently reported [26]. However, it has not been determined how visual/beta arrestins are related to similar proteins in fungi [27,28] and animals [31-33]. The official gene names of the mammalian proteins are Arrestin Domain Containing 1–5 (Arrdc1–5) and Txnip [nomenclature/aliases of vertebrate arrestins are discussed in Additional file 1]. We refer to that subfamily as alpha arrestins – to distinguish them from their close relatives, the visual/beta arrestins, and their more distant relatives, Spo0M and Vps26 (all of which contain arrestin domains, below). Here we report the evolutionary history of the arrestin clan. Those findings made us consider whether visual/beta arrestin emerged in concert with opsin. That, in turn, led us establish that opsins predated Bilaterata (also see [34,35]). We discuss what our results suggest about eye evolution and arrestin functions.

## Results and discussion

### Identification of novel members of the arrestin clan

**Figure 1:**
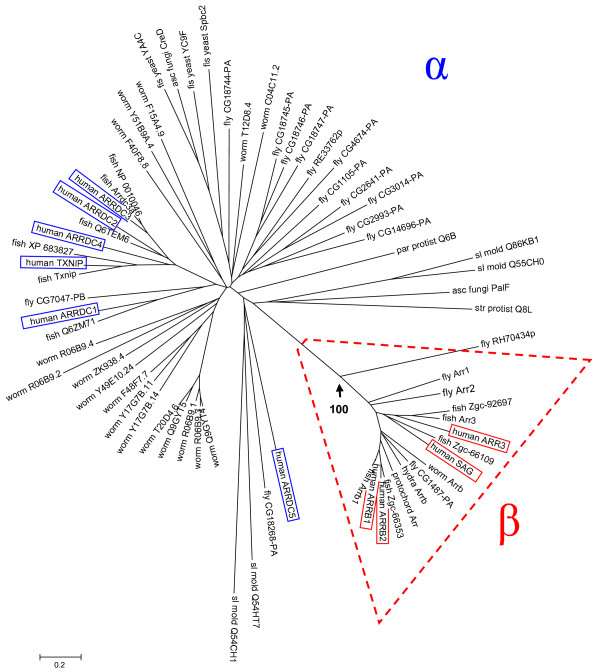
**Arrestin family tree**. Annotated arrestin proteins from select sequenced genomes were used to construct a neighbor-joining phylogenetic tree. The visual/beta arrestin proteins branch from the remainder of the tree with a bootstrap confidence score of 100 (arrow). The scale bar shows the number of substitutions per site. Taxonomy abbreviations follow: str_protist, stramenopiles 9STRA (Protista, Stramenopiles); par_protist, *Paramecium tetraurelia *(Protista, Alveolata); sl_mold, slime mold *Dictyostelium discoideum *(Protista, Mycetozoa)*; *fis_yeast, fission yeast *Schizosaccharomyces pombe*; asc_fungus, *Emericella nidulans *(Fungi/Ascomycota); hydra, *Hydra magnipapillata *(Cnidaria); worm, nematode *C. elegans *(Nematoda); fly, *Drosophila m*. (Arthropoda); protochord, *Ciona intestinalis *(Urochordata); fish, *Danio rerio *(zebrafish; Vertebrata). Vertebrate visual/beta arrestins are given in HUGO nomenclature [see Additional file 1]: S-antigen, SAG (aka, rod arrestin, arrestin 1); arrestin 3, ARR3 (aka, cone arrestin, X-arrestin, arrestin 4); arrestin, beta 1, ARRB1 (aka, arrestin 2); and arrestin, beta 2, ARRB2 (aka, arrestin 3).

**Figure 2:**
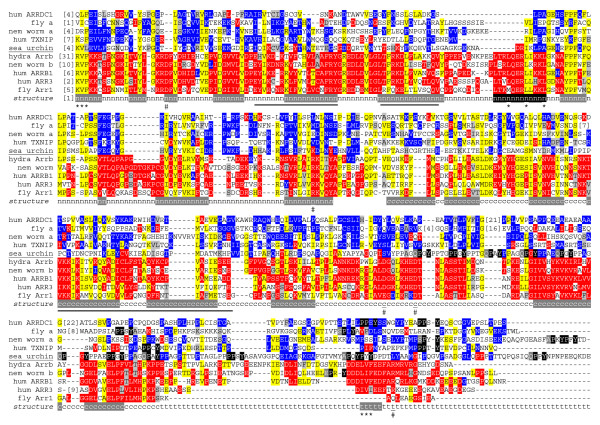
**Arrestin protein family: multiple sequence alignment of phylogenetically diverse members**. The alpha and beta classes of arrestins are distinct. Positions that may be widely conserved by common descent are yellow, beta/visual-specific are red and alpha-specific are blue (ambiguous in gray). Black positions in the Tail are the PPXY (or (P/L)PXY or "PY") motifs. PPXY motifs can have alternative residues in the first position. Notably, the sea urchin alpha arrestin has two PPXY and seven QPXY motifs. The sea urchin and nematode alphas also share the PY-like sequence (Y/F)APXYP(Y/F)Y. The arrestin domains are given below the alignment, n for N domain, c for C and t for Tail; italics show sequence not considered as part of the N and C domains according to Pfam. Shading on that line maps secondary structure elements on cone arrestin (symbol ARR3, HUGO nomenclature; aka arrestin 4, X-arrestin) – beta strands in gray and the one alpha helix in black [78]. Underlining highlights regions involved in receptor specificity, as described in Ref. [78] and references within. Two sets of intra-molecular interactions are important for keeping visual/beta arrestins in their basal conformation (see text): number symbols (#) mark residues that make up the "polar core"; asterisks (*) show residues involved in the "three-element interaction". Identifiers follow: fly a, *D. melanogaster *alpha arrestin CG18745-PA; nem worm a, nematode *C. elegans *alpha T12D8.4; nem worm b, beta F53H8.2; sea urchin, *Strongylocentrotus purpuratus *alpha XP_001175756; others are listed by gene name [see Additional file 2].

We conducted phylogenetic analysis of arrestins and found a large tree of mostly unrecognized arrestins in eukaryotes (Figs. 1, 2) [see Additional files 1, 2, 3, 4]. Figure 1 is a phylogenetic tree of arrestins from select evolutionarily diverse genomes. It shows that all the known animal arrestins [26] are members of a small branch of the protein family that emerged relatively recently. We suggest that the two subfamilies could be termed alpha and beta (or visual/beta) class arrestins [discussed in Additional file 1]. This applies because the alphas are the ancient/ancestral arrestins and it fits well with the historical name of the betas (named in reference to beta adrenergic receptors).

We find varied numbers of arrestins in the phyla previously known to have arrestins – fungi and animals. For example, the yeast *Schizosaccharomyces pombe *has three arrestins. Nematode worms and flies each have approximately twenty. Half of the worm and fly arrestins appear to have emerged in their respective lineages. Humans have a total of 10 arrestins. For comparison, humans have approximately the same number of 7TMRs as *Caenorhabditis elegans *and four times as many as *Drosophila *[4]. Even protists, which are single-celled, show diverse numbers of arrestins [see Additional file 3]. For example, *Dictyostelium*, which has approximately 55 7TMRs [36], has four arrestin genes, and *Tetrahymena *has *fifteen*. It is worth noting that *Tetrahymena *have much more complex membrane and cytoskeletal architecture than animals. Their genome has a striking expansion of genes involved in membrane and cytoskeletal dynamics [37]. Moreover, the percent of their genome devoted to kinases is double that of fungi and animals, but they only have four annotated 7TMRs. This hints that early arrestin function could have been associated with membrane/cytoskeletal dynamics or kinase signaling.

**Figure 3:**
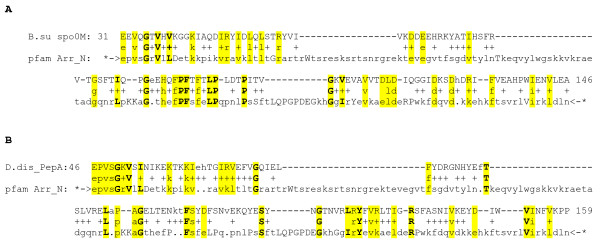
**Bacterial Spo0M and Eukaryotic Vps26 proteins are members of the arrestin clan**. (A) Sequence alignment of *B. subtilis *Spo0M and Arrestin N domain consensus (Pfam HMMER, statistical significance score E = 9.9e-5 [see Additional file 2]). (B) Alignment of *Dictyostelium discoideum *PepA (Vps26) and Arrestin N domain (E = 0.02). The Pfam motif pattern includes weakly (lower case) and highly conserved positions (bold upper case). Conserved sequence is yellow.

Compared to visual and beta arrestins, alphas have conserved protein sequence and domain topology spanning almost their full lengths. The high sequence conservation suggests their molecular functions are similar. For example, human TXNIP matches Arrestin N and C domains with Expect scores of 3.3e-46 and 9.8e-28, respectively (using HMMER, see Methods). We also found two domains that appear to be distantly related to arrestins, Spo0M and Vps26 (Fig. 3). Both are clearly alpha arrestin-like, rather than beta. This finding was independently made by investigators managing the Pfam database of curated protein patterns and posted on their database (Accession CL0135; J. Mistry, unpublished). They validate this "Arrestin N-like clan" with one multiple sequence alignment of most available members of the three protein subfamilies, and provide confidence scores of 1e-9 (arrestin N-Spo0M) and 2e-8 (arrestin N-Vps26).

Spo0M proteins are present in archaea (at least in multiple species of Halobacteriaceae) and in diverse bacterial phyla (Cyanobacteria, Actinobacteria, Gamma/Beta-proteobacteria and Firmicutes), but not in eukaryotes. Notably, archaea and bacteria have seven transmembrane proteins (structurally related to eukaryotic 7TMRs) and serine/threonine/tyrosine-specific kinases, both of which were widely believed to be eukaryotic inventions until recently [7,38]. Little is known about Spo0M except that it is transcriptionally regulated by sigmaH and that it inhibits sporulation. It is tempting to speculate that Spo0M has a role in transmembrane signaling that links environmental status to sporulation.

The crystal structure of a mammalian Vps26 protein revealed that it has two arrestin domains [39,40]. However, it was concluded there is no protein sequence similarity, presumably because Vps26 was compared to the visual/beta arrestins (and not to the much more closely related alpha arrestins) [40]. It was also suggested that the structural similarities between the two subfamilies are mostly superficial [39]. The claim is that Vps26 lacks all of the specific functional features of visual/beta arrestins: mainly, the sites responsible for GPCR, clathrin/AP2, and phosphatidylinositol phospholipid binding. Two groups disagree on the possibility that the two proteins could have similar switch mechanisms based on conformational rearrangements [39,40]. While both proteins have polar and electrostatic interactions between the N and C domains, they are mediated by different residues on different secondary structural elements. A caveat is that this analysis is limited by the absence of structural and functional information about alpha arrestins. That is, Vps26 is being compared to distantly related visual/beta arrestins, rather than the much more closely related alpha arrestins. Our results, and those of Pfam, indicate Vps26 and arrestin are true homologs. Alpha arrestins have robustly similar structure predictions to those solved for beta class arrestins and Vps26 [23,40,41] [see Additional files 1, 4]. For example, "threading" of alpha arrestin TXNIP through all known crystal structures matches rod visual arrestin and Vps26 with an identical probability value of 1e-10 (using GenThreader [see Additional file 2].

The Vps26 domain appears to be present in all eukaryotes (see Pfam PF07070). Its presence in plants is significant because they lack arrestin. Similar to arrestins, Vps26 proteins very rarely have associated domains. In addition to the two known human Vps26 genes – *VPS26 *and *VPS26B *– we found two new ones: *DSCR3 *and an un-annotated gene on 2q33.3 (Methods). Vps26p was discovered in a genetic yeast screen for "vacuole protein sorting, Vps" [42]. The yeast vacuole is the equivalent of the mammalian lysosome. Vps26 forms part of the retromer complex that mediates protein transport from endosomes to the trans-Golgi [43]. It is also involved in receptor transcytosis in polarized cells as well as in Wnt signaling and gradient formation [44]. The retromer has two subcomplexes, one responsible for cargo-selection and the other for vesicle formation. Vps26, Vps35 and Vps29 form the receptor cargo-binding subcomplex. Vps26 also has an adaptor role linking that subcomplex to the membrane-binding subcomplex. However, the exact biochemical role of Vps26 is not defined. While there are four Vps26 genes in mammals, the other retromer components are represented by single genes.

Retromer function is thus reminiscent of the role of beta arrestins in receptor endocytosis. Both arrestin and Vps26 are part of a transmembrane receptor-binding complex on the cytoplasmic face of endosomes. Arrestin further interacts with the membrane via the clathrin adaptor AP-2, and Vps26p does so through Vps5p/Vps17p (referred to as sorting nexins 1/2 in mammals). Both arrestin and Vps26 form complexes that co-localize with key vesicular trafficking proteins such as Rab5 and N-ethylmaleimide-sensitive factor (NSF). NSF interacts directly with beta arrestin [45] and is required for retromer endosome-to-golgi trafficking [46]. It is curious that beta arrestins bind the Vps26 partner Vps35 [47], and that Vps35 is present in 7TMR complexes [48]; however, there is no evidence those interactions are biologically relevant.

### Alpha arrestins were present in early eukaryotes

We used PSI-BLAST to search for all arrestins in the GenBank protein database (Methods). We found alpha arrestins in fungi and in all multi-cellular life *except plants*. Mammals have six alpha and four visual/beta arrestins [see Additional files 1, 4]. The significant protein-sequence difference between some of those alpha arrestins suggests they have more diverse functions than the more closely related visual/betas. The single arrestin present in viruses is a vertebrate alpha arrestin, *Arrdc3*, that was horizontally transferred to canarypox virus [Additional files 1, 5]. We also identified arrestins in all protist groups except Rhizaria, which has little sequence available. In Additional file 3 we describe the protist proteins. Since Arrestin N and C domains are related by protein sequence, we searched for single-domain proteins that could be the ancestral arrestin domain that was duplicated. We found that no arrestin-containing kingdom or species has exclusively single-domain proteins. This result is consistent with the finding that the VPS26 crystal structure reveals tandem domains [40]. We believe a twin domain gene was duplicated in a bacterium or basal eukaryote and gave rise to genomes with both arrestin (which was subsequently lost in plants) and Vps26.

We searched for arrestin-associated domains that could hint at biochemical functions. Outside of the protists, we found that these are extremely rare. We found exactly two domains that recurred in at least two divergent phyla; they were in two distantly related orders of the fungus-like protists Amoebozoa [see Additional file 3]. Two *Dictyostelium *and two *Entamoebidae *arrestins each contain a C2 domain, which is a Ca^2+^-dependent phospholipid/membrane binding element. In one order the C2 domains are N-terminal in both proteins, in the other both are C-terminal. This suggests they could have been created by independent events of exon shuffling in the two orders. Two other genes, one from each of the same two orders, contain one FYVE domain in their C-terminal region. FYVE domains have exquisite specificity for phosphatidylinositol-3-phosphate (PtdIns(3)P), an endosomal marker recognized by proteins involved in signaling and trafficking. Notably, Vps26 interacts with the PtdIns(3)P-binding protein sorting nexin 1 [49]. The presence of associated C2 and FYVE domains indicates some ancient arrestin functions involve plasma and endosomal membrane interactions, respectively. This is consistent with known arrestin roles in transmembrane receptor-binding and endocytic trafficking.

### Difference between alpha and visual/beta arrestins

Figure 2 and Additional file 4 show multiple sequence alignments comparing diverse known and new arrestins. While many positions are conserved in all arrestins, it is clear that there are two classes of arrestins – the visual/beta class and the more ancient alpha class. The protein analysis is discussed in Additional file 1. The most salient features of this comparison are 1) alpha arrestins lack the arrestin N domain helix [see Additional files 4, 6], and 2) alphas, but not visual/betas, have PPXY (or (P/L)PXY, "PY") motifs (Fig. 2, Additional file 4).

Helix I of visual/beta arrestins is sequestered in the inactive conformation and is presumably released upon activation [41]. This helix has hydrophobic residues on one face and basic residues on the other. However, it is a mystery what it does, or interacts with, in the active conformation. Helix I leucine substitutions suggested a role in receptor binding [25], and it was later theorized to be a membrane-docking element that permits non-specific interactions with activated receptors [41]. Other experiments showed that helix I is important for the formation of dense-core vesicles [50]. The proposed membrane docking role of helix I remains attractive to us. We add that such an insertion would displace membrane on the cytoplasmic leaflet of the plasma membrane. This could induce positive curvature and promote endocytocis. Notably, helix I is absent in alpha arrestins (Fig. 2, Additional files 4, 6) and is thus a major innovation in beta arrestins. That interpretation is supported by the absence of helix I in the 3D structure of VPS26 [40], which has protein sequence similarity to alpha arrestins.

All indications suggest apha and beta arrestins have similar structural topologies, *but do alphas also bind 7TMRs? *Nichols and Sanders-Bush announced their discovery of a new mammalian arrestin (now alpha) in 2004 [31]. However, to our knowledge there are no published studies of arrestin-like functions in animal alpha arrestins. The current understanding of alpha arrestin biochemistry thus comes from fungi and yeast. Herranz, Vincent and colleagues showed that fungal PalF (*Aspergillus nidulans*) is a bona fide arrestin by protein sequence and function [28]. PalF binds C-terminal sites of the activated seven transmembrane pH sensing receptor PalH. Moreover, alkaline activation induces PalH-dependent phosphorylation and ubiquitination of PalF. Truncation of the PalH cytoplasmic domain disrupts PalF-binding and inhibits growth in alkaline pH. The alpha arrestin PalF thus resembles beta arrestins in its ability to bind active receptors, generate a signal and be posttranslationally modified in the process. The function of PalF, however, is in signal transduction and apparently not in inhibition of G protein signaling. pH sensing and "vacuole protein sorting, Vps" pathways are intimately associated in fungi. Herranz et al. thus propose that the role of PalF is likely to relate to endocytic trafficking. This is notable considering that Vps26, an ancient arrestin relative, was discovered in a genetic screen for Vps genes.

While beta arrestin tail domains have conserved clathrin-interacting motifs, alphas have PY motifs. *Is there evidence the latter are functional? *PY motifs bind WW domains and their interactions are extensively defined by diverse biochemical and structural methods [51]. The strong conservation of multiple PY motifs in fungal and animal arrestins suggests they interact with WW proteins. *Saccharomyces cerevisiae *has three alpha arrestins, all of which have turned up in biochemical and genetic screens [see Additional file 1]. Without knowing they were studying arrestins, others showed that two *S. cerevisiae *arrestins (Rod1p and Rog3p) bind the HECT (or Nedd4 family) E3 ubiquitin ligase Rsp5p [52,53]. Moreover, they showed by mutagenesis that the interactions are mediated by two PY motifs in arrestin and two WW domains in Rsp5p [52]. Our computational analysis of the human genome identified 25 WW proteins that may interact with alpha arrestins (not shown). Notably nine of those genes are candidate co-orthologs of yeast Rsp5p. This suggests alpha arrestins could be involved in ligand-dependent 7TMR ubiquitination and trafficking [18,54], which, in some yeast and mammalian cases, are mediated by HECT E3 ligases [55,56]. We also suspect arrestin PY motifs interact with other WW proteins that are involved in cytoskeletal dynamics, endocytocis/trafficking and signaling. Using yeast two hybrid methodology, we have found that alpha arrestin tails robustly bind the WW domains of HECT E3 ligases and of other proteins (F.-C. Hsieh, W.-K. Chen and C. A., unpublished).

### Origins of visual/beta arrestin and rhodopsin

**Figure 4:**
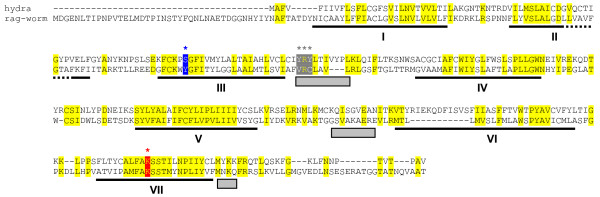
**Novel *Hydra *opsin aligned with rag-worm ciliary opsin**. As expected, the sequence conservation is highest in the seven transmembrane regions (underlined). The signature residue perfectly conserved in all opsins is the lysine that retinal forms a Schiff base with. This conserved position is shown in red. The position of the well-characterized glutamate counterion of some ciliary opsins, is instead a tyrosine in several ciliary opsins and all rhabdomeric opsins. *Hydra *ciliary opsin has a serine at that position. The cytoplasmic regions shown by mutagenesis to be critical for G protein interaction are underlined by gray boxes. The first of those cytoplasmic regions contains the highly conserved DRY element, which contains an arginine critical for G protein activation. That sequence is more similar in *Hydra *(YRY) than in rag-worm (VRC), shown by gray highlighting.

The fact that all known visual arrestins cluster together with beta arrestins suggested to us that this branch may have emerged together with an early opsin. We used visual/beta arrestin and visual/beta arrestin consensus sequences to search GenBank for the earliest family member we could find (Methods). The oldest visual/beta arrestin we found is from *Hydra *(Cnidaria; Figs. 1, 2). Notably, Cnidarians are the only beta arrestin-containing organisms that predate any known opsin. We next searched for the oldest opsin genes present in all, but the Trace database of GenBank (Methods). That analysis resulted in the identification of multiple *Hydra *ESTs encoding partial opsin proteins (data not shown). Of the known subfamilies, we only found representation of ciliary opsin. We were able to assemble the full length open reading frame for one of these (Fig. 4; Methods). This Cnidarian protein is strikingly similar to ciliary opsins, placing it in the same group as rag-worm ciliary opsin (non-visual [57]) and human visual opsins on the phylogenetic tree of opsins [10].

Cnidarian opsin genes are the first to be reported that predate the emergence of Bilaterata [57]. While the present study was under review, others independently reported finding Cnidarian opsins [34,35]. As we did, both of those groups found that Cnidarians have ciliary opsins. They additionally reported other highly divergent opsins that cannot be clearly classified according to the known subfamilies. Remarkably, Suga, Schmid and Gehring identified large numbers of opsin genes in single species of Cnidarians: 30 sea anemone, 63 hydra, and 18 jellyfish [35]. They showed jellyfish opsins are expressed in multiple patterns, including in the eyes and gonads (similar to a previous finding in fruit fly testes [58]). Neither those studies nor ours (data not shown) were able to find any evidence of opsins in sponges or older phyla. This is consistent with the fact that sponges use flavin or carotenoid photopigments [59].

The early evolution of vision is an open question [9]. It was long known that some jellyfish have image forming eyes, but the molecular identity of their visual pigments was a mystery until now. Recently it was postulated that the absence of a master transcriptional regulator, *Pax6*, in the box jellyfish is consistent with the independent evolution of eyes in higher metazoans [60]. Gehring [9] offered two alternative explanations to that same question: vertical evolution from a photosensitive protist and horizontal evolution through a photosynthetic cyanobacterial symbiont. We proposed that the phylogeny of the phototransduction machinery would explain the evolution of phototransduction and vision [61]. The new opsin phylogeny suggests to us that ciliary opsins are likely to be ancestral to all previously known [10] visual pigments in animals. That possibility is consistent with Darwin's theory that eyes evolved once [8]:

*How a nerve comes to be sensitive to light hardly concerns us more than how life itself originated; but I may remark that, as some of the lowest organisms in which nerves cannot be detected are capable of perceiving light, it does not seem impossible that certain sensitive elements in their sarcode should become aggregated and developed into nerves endowed with this special sensibility*...

*The simplest organ which can be called an eye consists of an optic nerve surrounded by pigment-cells and covered by translucent skin, but without any lens or other refractive body. We may, however, according to M. Jourdain, descend even a step lower, and find aggregates of pigment-cells, apparently serving as organs of vision, without any nerves, and resting merely on sarcodic tissue. Eyes of the above simple nature are not capable of distinct vision, and serve only to distinguish light from darkness*.

The fact that both opsin and visual/beta arrestin are present in Cnidaria, but not older phyla, hints that the two subfamilies could have emerged in concert. Early phototransduction offered major advantages, such as photoperiodicity and phototaxis. Presumably, the first step was the creation of photosensory opsin. However, there must have been immense avenues for improvement after that point. One challenge to early opsin was exposure to light intensity that varies by 10 orders of magnitude, the earth's daily illumination cycle. The innovation in visual/beta arrestin could have improved the modulation of signaling gain by regulating opsin localization, endocytosis/recycling or inactivation kinetics. Gene duplication is a hugely important mechanism for evolution [62]. Visual phototransduction consistently uses dedicated proteins created by gene duplication (best characterized in flies and mammals [63,64]). For example, in addition to opsin 7TMRs, mammalian photoreceptor cells have their own, or nearly exclusive, G proteins, cGMP-phosphodiesterases, G protein-coupled receptor kinases, arrestins, etc. Outside the eye, a handful of each of those proteins transduces signals for the remaining hundreds of 7TMRs. This extreme specialization illustrates the high importance of vision in animal evolution.

### Possible implication of pre-Cambrian vision

Both opsin and visual/beta arrestin are present in Cnidaria, but not in sponges, the most basal metazoans (nor in any older model organism). This is interesting in light of Parker's recent proposal that vision set off the Cambrian explosion of life forms [65]. 543 million years ago (MYA) only three phyla existed, but, in the following 5 MY, 35 phyla emerged. No new phyla have emerged since that *Cambrian explosion*. [This is widely accepted, but some have argued it may be an artifact of the fossil record [66].] Parker proposed that vision triggered the Cambrian explosion by creating a new world of organismal interactions [65]. The key observation is that pre-Cambrian phyla were soft-bodied. However, the Cambrian saw an apparently limitless diversification of hard body parts. At the same time, the use of biological color appeared. Parker claims this scenario can be explained by the emergence of vision, which must have resulted in new behaviors such as predation. Seeing-predators would have suddenly needed rigid components to pursue, attack, and eat their prey (e.g., in limbs, jaws, and sharp mouth parts). Their prey – which may or may not have had eyes – also had to adapt by developing hard shells or spines, camouflage or even invisibility (as is seen in jellyfish).

Parker specifically hypothesized that this "Light Switch" was triggered by the emergence of arthropod eyes during the Cambrian. Put another way, he proposed the Light Switch required image perception and brain processing of the information (i.e., this was The trigger, and precursors of such vision did not provide a major evolutionary stimulus). We find his general argument to be compelling. However, we believe the spark had to *precede *the Cambrian explosion. We propose that light perception in a pre-Cambrian animal initiated new dimensions of organismal and environmental interactions. This may have progressed steadily until a critical mass of interactions was reached, the Cambrian explosion. Our findings (and also [34,35]) are clear evidence that opsin (and beta arrestin) predated the Cambrian period. This meets what we consider to be the first requirement for a visual trigger hypothesis. If sight initiated an arms race in the biosphere, it is not surprising that some organisms refined it and others lost it (e.g., some predators and prey, respectively). Only six of 38 phyla on earth have eyes, but these six seeing phyla account for >95% of all living animals. It is not difficult to imagine that the relative success of seeing animals is due to the extraordinary advantage of vision.

## Conclusion

Despite the high interest in GPCR signaling, its evolution remains enigmatic. There is some evidence that archaeal and bacterial 7TMRs are homologous to eukaryotic 7TM/GPCRs [7]. However, heterotrimeric G proteins/G alpha subunits are only present in eukaryotes. This suggests that ancestral 7TM/GPCRs signaled by mechanisms other than G protein coupling. We found that the arrestin clan is present in archaea and bacteria, raising the possibility that Spo0M could be a primordial 7TMR signaling partner. In addition, our findings of Cnidarian opsins lead us to propose that the ciliary subfamily is ancestral to all bilaterian opsins (also see [34,35]). That is consistent with Darwin's theory that eyes evolved once.

There were two major arrestin-like gene families in early eukaryotes, arrestin and Vps26. Both protein families are well characterized and point to endocytosis/endosomal dynamics as the ancestral arrestin/Vps26 functions. The duplication of the arrestin domain was a critical event in the creation of ancestral arrestin/Vps26. This could have created autoinhibitory mechanisms (such as those seen in beta arrestins), a recurrent theme in the evolution of signal transduction. The functional similarities of beta arrestins and Vps26 proteins lead us to speculate that the original arrestin/Vps26 was involved in receptor internalization. This could have had two classes of receptor effects in concert: 1) desensitization and recycling/degradation, and 2) signaling. Others have hypothesized that the original role of arrestins may have been as signaling adaptors rather than terminators [67]. Above we mention biochemical evidence that mammalian Vps26 and arrestins could have overlapping roles [47,48].

The homology of alpha and beta arrestins suggests their molecular functions may be similar. There is evidence from fungi that the alpha arrestin PalF specifically binds an activated 7TMR [28]. That interaction has a positive signaling role that is not yet identified. There are also differences between the alpha and beta classes. Beta arrestins are generally cytoplasmic in unstimulated cells, while alpha arrestins are often associated with membranes [32,68]. Only visual/betas have helix I in the N domain. And the tails of betas contain clathrin-interacting motifs, while those of alphas have PY motifs. Studies in yeast showed that alpha arrestin PY motifs interact with the WW domains of the HECT E3 ubiquitin ligase Rsp5p. We believe that a major role of alphas is to recruit WW proteins to activated receptors. Alpha and beta arrestins are widely co-expressed. The fact that visual/beta arrestins can hetero-associate [69], hints that alphas and betas may also. Given the near ubiquitious involvement of beta arrestins in 7TMR signaling, we speculate that alphas and betas may function coordinately.

## Methods

### Identification of alpha arrestins

In the course of conducting BLAST analysis [70] of GenBank for TXNIP-related proteins, we found it is a member of the arrestin protein family. We termed the new arrestin class the alpha arrestins. We confirmed our findings using the Pfam Hidden Markov Modeling HMMER server (Refs. [71,72]) to test for similarities to the their database of curated protein patterns [73]. TXNIP matches two domains with apparent statistical certainty: Arrestin N (Expect score, 3.3e-46) and Arrestin C (9.8e-28). Thus the similarity of alpha and beta arrestins is strong and can be detected by comparison of individual members (e.g., using BLAST) or by comparison to consensus sequences (Conserved Domain-Search, NCBI; [74]) and protein family alignment matrices (HMMER).

### Identification of Spo0M and Vps26 as arrestin clan members

Both these domains can be identified using domain searching programs such as HMMER and Conserved Domain-Search (NCBI; [74]) with a subset of arrestins. The resulting Vps26 and Spo0M hits match only the arrestin N and C domain regions of arrestins. We also tested the families with Position-Specific Iterated (PSI) BLAST (NCBI; [75]), which uses multiple search iterations to "learn" a pattern from related sequences (building a score matrix after each round). PSI-BLAST analysis of all GenBank proteins with single members of the two families exclusively yields the known family members on the first "iteration" (the initial search). The second iteration of the Vps26 search yielded several insect, nematode and vertebrate arrestins above the program's confidence threshold (the best Expect score being for *C. elegans *F58G1.6, 3e-04). The third iteration of the Spo0M search also identified insect, nematode and vertebrate arrestins (the best score being *C. elegans *F48F7.7, 1e-09). In addition to the two known human Vps26 genes – *VPS26 *and *VPS26B *– we found two new ones: Down syndrome critical region protein 3 (*DSCR3*) and what appears to be another intron-containing active gene on 2q33.3 (including 207,259,576-207,260,296 on the UCSC Mar. 2006 assembly).

### Hydra beta arrestin and opsin discovery

We used beta arrestin, beta arrestin consensus sequences, and diverse opsin proteins to BLAST GenBank protein and DNA databases (translated in all frames). The only pre-bilaterian beta arrestin and opsin were found in the *Hydra *EST DNA database. The opsin was found by querying with rag-worm ciliary opsin, which was the most ancient known opsin [57]. Many *Hydra *beta arrestin and c-opsin ESTs were identified and the coding cDNA sequence was unambiguously assembled from several highly overlapping ESTs. The EST assemblies with accession numbers and the translated protein sequence are given in Additional file 2. Curiously, Santillo and coworkers recently cited a *Hydra *Expressed Sequence Tag (EST) annotated as being similar to mouse peropsin [76]. We now find that EST (which was only mentioned by accession number, but not described as DNA or amino acid sequence) is one of those we used to assemble the *Hydra *opsin cDNA. [That EST, CB073527, is dated from 2003 in GenBank and attributed to H. Bode et al., Washington University Hydra EST Project.]

### Phylogenetic tree building

Protein sequences were aligned using Clustal W. Manual corrections were made and gaps were trimmed. A neighbor-joining tree was made from amino acid pairwise distance with Poisson correction (MEGA2, [77]). The bootstrap value of the beta class branch was calculated from 1000 repetitions. A similar score is seen for the Maximum Parsimony tree. Organism abbreviations followed by the common name given on Fig. 1, in parentheses are provided below. The available sequence names and accession numbers are given in Additional file 2. Protein sequences assembled with high confidence from GenBank DNA sequences are provided with DNA sequence annotations in Additional file 2. Taxonomy abbreviations follow: fish, *Danio rerio *(zebrafish; Vertebrata); protochord, *Ciona intestinalis *(Urochordata); fly, *Drosophila m*. (Arthropoda); worm or nem_worm, *Caenorhabditis elegans *(Nematoda); sea urchin, *Strongylocentrotus purpuratus *(Echinodermata); hydra, *Hydra magnipapillata *(Cnidaria), par_protist, *Paramecium tetraurelia *(Alveolata); asc_fungus, *Emericella nidulans *(Fungi/Ascomycota); fis_yeast, *Schizosaccharomyces pombe *(Fungi/Ascomycota); str_protist, stramenopiles (Protista); sl_mold, *Dictyostelium discoideum *(Protista, Mycetozoa). Sequence accession numbers are provided in Additional file 2.

## Authors' contributions

All work was done by the author.

## Supplementary Material

Additional file 1 **Supplementary text**. A Doc file with discussion of arrestin protein sequence analysis, comparison of human and fish arrestin proteins, human complement of arrestin genes, and the horizontal gene transfer of vertebrate *Arrdc3 *to a poxvirus. Click here for file

Additional file 2 **Supplementary methods**. A Doc file with the methods used in the discovery and analysis of protist arrestins, discovery and protein sequence alignment of zebrafish and human arrestins, secondary and tertiary structure predictions of alpha arrestins, and phylogenetic analysis of canarypox virus arrestin. All protein sequence annotations are also provided here. Click here for file

Additional file 3 **Table of protist arrestins**. A PDF file with Table 1. Details of protist arrestins. Click here for file

Additional file 4 **Multiple sequence alignment of human and zebrafish arrestins**. A PDF file showing a multiple sequence alignment of the human arrestin family and select fish arrestins. It shows protein conservation and divergence of different vertebrate arrestins. Alignment region of beta arrestin helix I suggests this structural element is absent in alpha arrestins. The conservation of PY motifs in alpha arrestins suggests they are functional. Click here for file

Additional file 5 **Phylogeny of canarypox virus alpha arrestin**. A PDF file showing the phylogeny of canarypox virus (CNPV) alpha arrestin. This analysis shows CNPV arrestin is a highly divergent vertebrate Arrdc3 that was acquired by horizontal gene transfer. Click here for file

Additional file 6 **Helix I region in vertebrate arrestins**. A PDF file showing evidence that helix I of visual/beta arrestins is absent in alpha arrestins. Protein sequence alignment and secondary structure prediction of alpha arrestins suggests they lack the helix I present in beta arrestins. Click here for file
