# Supplementary material for: On the origins of arrestin and rhodopsin
Source: BMC Evol Biol. 2008 Jul 29;8:222. doi: 10.1186/1471-2148-8-222 (PMC2515105; doi:10.1186/1471-2148-8-222)
Supplement: Additional file 1 — Supplementary text. A Doc file with discussion of arrestin protein sequence analysis, comparison of human and fish arrestin proteins, human complement of arrestin genes, and the horizontal gene transfer of vertebrate Arrdc3 to a poxvirus. [file 1471-2148-8-222-S1.doc]

**Additional file 1. Discussion**

**Nomenclature**

Throughout this work, we use official HUGO nomenclature names/symbols [1]. Each visual/beta arrestin has multiple aliases. These are the official HUGO terms, followed in parentheses by aliases from the NCBI OMIM and Gene databases, and from a “systematic nomenclature” (apparently based on order of discovery): S-antigen, SAG (aka, rod arrestin, S-arrestin, retina and pineal arrestin, arrestin 1); arrestin 3, ARR3 (aka, cone arrestin, (retinal) X-arrestin, arrestin 4); arrestin, beta 1, ARRB1 (formerly arrestin 1, ARR1; aka, beta-arrestin 1, ARB1, arrestin 2); and arrestin, beta 2, ARRB2 (formerly arrestin 2, ARR2; aka, beta-arrestin 2, ARB2, BARR2, arrestin 3). The major aliases for alpha arrestins follow: ARRDC2 (ILAD-1), ARRDC3 (TLIMP, KIAA1376), ARRDC4 (DHR1), and TXNIP (VDUP1, TBP2).

Protein families and subfamilies are generally defined by protein sequence conservation. Our nomenclature here is consistent with existing databases of protein patterns, which classify arrestin, Vps26 and Spo0M as protein families. Additionally, Pfam coined the term arrestin clan, which includes all three families. We confirm that the arrestins have a protein sequence signature that distinguishes them from all other proteins, including Vps26 and Spo0M. Moreover, we show that arrestins make up two branches with their own protein sequence signatures (Figs. 1, 2; Additional file 4). We refer to one subfamily as beta or visual/beta class, and the other as alpha class. Alpha fits because this subfamily is ancient/ancestral, and it complements the name of the beta class. [Since there are other proteins with arrestin domains (Vps26 and Spo0M), it would be problematic to refer to the new subfamily as “arrestin domain containing”, “arrestin-like”, or “proto-arrestin”. Moreover, there is insufficient knowledge to consider the use of functional terms such as receptor-binding or sensory arrestin.]

**Protein Sequence Analysis of Alpha Arrestins**

Alpha and beta arrestins have robust conservation of Arrestin N and C domains. The sequence P(E/D)XXXQP in the 11 amino acid hinge of beta class arrestins is also widely conserved in alphas. The C terminus of the beta/visual arrestins is comprised of beta strand XIX (coordinated with the C domain) and a long tail that lacks secondary structure except for the small beta strand XX near the end [2]. Thus most of the tail lacks secondary structure. However, it has tertiary structure in the inactive conformation due to several interactions with parts of both the N and C domains. Based on our secondary structure predictions, alpha arrestins share the lack of secondary structural elements over most of the Tail (T) domain. We find that this tail region is a true protein domain. T domains may be very weakly conserved throughout arrestin evolution, but they have clear fingerprints specific to alpha and beta classes. The distinct alpha and beta T domain patterns are well conserved in humans and fish, which diverged approximately 500 MYA [see Additional file 4].

Two components of the mechanism that maintains arrestin in the basal/inactive form are the dispersed “polar core” residues [2] and the “three element” interaction [3]. The three element interface is made up of the interacting C terminal tail beta strand XX, beta strand I and helix I. When arrestin is activated, the buried C tail is released [4] to interact with other proteins, such as clathrin components. The best-known active-conformation payload in the T domain of beta arrestins is the clathrin-binding motif. This motif is not present in the alpha arrestins. However, diverse sequences without that motif may bind clathrin – provided they have an appropriate combination of acidic/hydrophobic residues and conformation [5]. The human and fish alpha arrestins TXNIP, ARRDC2 and ARRDC3 have a cluster of acidic and hydrophobic residues in a similar position (Fig. 1). The other well characterized interaction of this region is with beta(2)-adaptin (the beta-subunit of the clathrin adaptor AP2). Six residues have been shown to be important for this interaction, five of which are conserved in beta arrestins. The protein alignment in that region is likely to be spurious, but we do not see similar sequence in the same vicinity of the alphas.

Beta strand XX of the three element interface has a conserved Ile/Val-Phe pair in the beta arrestins. We find such a conserved element slightly upstream of that position in TXNIP and ARRDC 2, 3, and 4 (although the alignment in that region is imprecise), and similar pairs or triplets in the same region of the other vertebrate alphas (Fig. 1, Additional file 4). In beta arrestins, that strand is immediately flanked by several acidic residues and followed by a conserved sequence known to interact with beta(2)-adaptin; both of those contextual elements are absent in alphas. We also find most of the positions that make up the “polar core” in betas are not conserved in alphas. However, those are single residues at diverse locations in the protein. A similar mechanism may be conserved, but mediated by polar residues at other positions. This is consistent with the similar crystal structures of visual/beta arrestins and Vps26 [6], which is much more closely related to alpha arrestins than visual/betas (but see [7]).

A reviewer of this work suggested that conservation of a polar core mechanism could be associated with a somewhat fixed distance between Arrestin N and C domains. Vertebrate alpha and visual/beta arrestins not only appear to have similar distances [see Additional file 4], but they also have a conserved amino acid motif in this hinge region (see above). In protists, the most ancient and diverse eukaryotes, one cannot precisely predict the boundaries of N and C domains. However, allowing for minor boundary errors, we predict that 23/27 twin domain arrestins have similar distances to those of vertebrates [Additional file 3]. Three of the four remaining proteins have dramatically greater distances, but they are all from two orders of the highly divergent Kinetoplastida (notably, they may also be unique in having single domain arrestins). The last atypical arrestin, which has an intermediate distance, is one of the four in *Dictyostelium*. It thus appears that arrestin hinge lengths are generally conserved across vast evolutionary time. Although this is consistent with constraints from a cooperative mechanism between N and C domains, there are other possibilities.

**Comparison of Fish and Human Arrestins**

Additional file 4 shows all human arrestins aligned with most zebrafish arrestins [see Additional file 2 for methods]. The only human members not found in fish are the alpha arrestins *ARRDC4* (identified in chick, XP_413881.2, but not in frogs) and *ARRDC5* (identified in chick (un-annotated, not shown) and mammals). Fish have at least 2 *Txnip*, 3 cone photoreceptor visual arrestins (*Arr3*) and *2 beta-Arrestin 2*. Along with a well conserved ortholog of *ARRDC1*, fish have three other *Arrdc1*-like genes. Notably, these counts are minima, as some fish genes (mainly those recently created by duplication [Additional file 2]) were omitted from the analysis. The last common ancestor of fish and humans existed about 500 MYA, corresponding to 1 BY of evolution between orthologous sequences. The amino acid conservation is high, indicating the proteins were already well perfected in the common ancestor. Remarkably, in the evolutionarily-tumultuous time since then (e.g., involving the move to land and the accelerated evolution of the brain), only two new arrestins were created in the lineage that includes chicken and human, *ARRDC4* and *ARRDC5*.

**Human Complement of Arrestin Genes**

The 10 human arrestins lie on 9 chromosomes (*ARRDC2* and *ARRDC5* are far apart on chr19). As with the protein sequence, the alpha and beta/visual arrestins can be easily distinguished at the DNA level. *ARRDC2/3/4* and *TXNIP* have the same gene structure with 8 exons. *ARRDC5* shares 2 of those 7 intron positions. *ARRDC1* shares 5 of the 7 intron positions, and has an additional pair of unique introns. The highly divergent and testis-specific *ARRDC5* is not well annotated in the human genome assembly; but it appears to only have 3 exons based on homology with the mouse cDNA. Each of the four beta and visual arrestins has 15 or 16 coding exons (both of the visual arrestins have one additional non-coding exon). The visual/beta arrestin-specific helix I is encoded by the middle of an exon. Downstream – but not upstream – of helix I, this exon has robust protein-coding conservation with alpha arrestins. This suggests helix I was created by sequence mutations or by upstream intron sliding (i.e., shifting of the 3’ splice site). It is also possible that a pre-existing helix from another gene was introduced by exon shuffling and that the new exon was subsequently fused with exon 3. The alpha arrestins are well below the average gene size of ~30 kb. They range from 3.9 kb (*TXNIP*) to 14.6 kb (*ARRDC3*) and average 10 kb. Beta and visual arrestins have both small genes (11 kb, *ARRB2*; 13.5 kb, cone arrestin *ARR3*) and medium/large (39.2 kb, rod arrestin SAG; 86.4 kb, *ARRB1*). *ARRDC2* is unique among the arrestins in that it has two alternative exons that encode the first half of the arrestin N domain (NCBI GeneID: 27106), and thus has two promoters.

**Saccharomyces Arrestin Interactions**

We searched the Saccharomyces Genome Database ([8]; http://www.yeastgenome.org/) to identify all known biochemical and genetic interactions of the three arrestins. We found that two, Rog3 and Rod1, have similar and overlapping biochemical interactions enriched for kinases. Rim8 is enrichment for direct interactions involved in DNA-related functions. Rim8 has ten known genetic interactions, but the others have none. The rim8 genetic interactions split into three categories – DNA-related, cell wall dynamics, and Class E Vps. The Vps group was uncovered by genetic rescue of three components – vps2 (did4), vps4 and vps24 – of the ESCRT-III complex, which is involved in the sorting of transmembrane proteins into the multivesicular body (MVB) pathway [9]. This is consistent with our hypothesis that the ancestral role of arrestins relates to endocytosis. Notably, the arrestin Rog3 is a substrate for phosphorylation by the GSK-3 family member Mck1 [10], which also interacts genetically with the arrestin rim8 [11]. This suggests arrestins may have a role in the endocytosis-related mechanisms of Wnt signaling that have recently gained attention [12]. Given that the retromer subunit Vps26 is a member of the arrestin clan, it is interesting that it is required – as are other retromer subunits – for Wnt signaling.

**Horizontal Transfer of Arrdc3 to a Poxvirus**

Poxviruses are prominent in medicine because one member, *Variola*/smallpox virus, has killed more humans than all other pathogens combined. Canarypox virus (CNPV) belongs to the avian poxviruses, the only genus of the vertebrate poxviruses that infects non-mammalian hosts. Poxviruses are notorious for having extensive gene acquisition that presumably relates to host-pathogen coevolution [13]. Sequencing of the CNPV genome revealed that it contains a gene related to vertebrate *Txnip* [14]. That gene is not present in other sequenced poxvirus genomes. Most importantly, this gene is absent in the closely related fowlpox virus genome. It was reported that the best match to one viral gene was human AB037797 (which corresponds to *ARRDC3*). However, the word arrestin does not appear in that publication. Instead, the horizontally acquired gene is discussed in light of its similarity to Txnip, a thioredoxin binding protein. We conducted phylogenetic analysis to identify the source of the acquired alpha arrestin, but can only say it was a vertebrate [see Additional file 5].

It is important to note that the gene gain was based on the original protein sequence. It is thus possible that *Arrdc3* was already important to CNPV – and other poxviruses – before the horizontal transfer. Unlike many other viruses, poxvirus tropism is not mediated by host-specific receptors. Instead, it seems to stem from a complex set of intracellular events that occur after binding/entry [15]. There are many pathways where arrestins could be involved, most obviously those relating to membrane receptors (e.g., the CNPV 7TMRs and host Toll-Like receptors involved in immunity), intracellular signaling (e.g., MAPK signaling involved in viral replication) and membrane trafficking (e.g., endocytosis, core uncoating and virion membrane wrapping/trafficking). Notably, *Vaccinia*, a close relative of smallpox virus, was recently shown to require clathrin-dependent endocytosis for viral spreading [16].

**References**

1. Eyre TA, Ducluzeau F, Sneddon TP, Povey S, Bruford EA, Lush MJ: **The HUGO Gene Nomenclature Database, 2006 updates**. *Nucleic Acids Res* 2006, **34**(Database issue):D319-321.

2. Hirsch JA, Schubert C, Gurevich VV, Sigler PB: **The 2.8 A crystal structure of visual arrestin: a model for arrestin's regulation**. *Cell* 1999, **97**(2):257-269.

3. Vishnivetskiy SA, Schubert C, Climaco GC, Gurevich YV, Velez MG, Gurevich VV: **An additional phosphate-binding element in arrestin molecule. Implications for the mechanism of arrestin activation**. *J Biol Chem* 2000, **275**(52):41049-41057.

4. Han M, Gurevich VV, Vishnivetskiy SA, Sigler PB, Schubert C: **Crystal structure of beta-arrestin at 1.9 A: possible mechanism of receptor binding and membrane Translocation**. *Structure* 2001, **9**(9):869-880.

5. Dell'Angelica EC: **Clathrin-binding proteins: got a motif? Join the network!** *Trends Cell Biol* 2001, **11**(8):315-318.

6. Shi H, Rojas R, Bonifacino JS, Hurley JH: **The retromer subunit Vps26 has an arrestin fold and binds Vps35 through its C-terminal domain**. *Nat Struct Mol Biol* 2006, **13**(6):540-548.

7. Collins BM, Norwood SJ, Kerr MC, Mahony D, Seaman MN, Teasdale RD, Owen DJ: **Structure of Vps26B and mapping of its interaction with the retromer protein complex**. *Traffic* 2008, **9**(3):366-379.

8. Nash R, Weng S, Hitz B, Balakrishnan R, Christie KR, Costanzo MC, Dwight SS, Engel SR, Fisk DG, Hirschman JE, Hong EL, Livstone MS, Oughtred R, Park J, Skrzypek M, Theesfeld CL, Binkley G, Dong Q, Lane C, Miyasato S, Sethuraman A, Schroeder M, Dolinski K, Botstein D, Cherry JM: **Expanded protein information at SGD: new pages and proteome browser**. *Nucleic Acids Res* 2007, **35**(Database issue):D468-471.

9. Hayashi M, Fukuzawa T, Sorimachi H, Maeda T: **Constitutive activation of the pH-responsive Rim101 pathway in yeast mutants defective in late steps of the MVB/ESCRT pathway**. *Mol Cell Biol* 2005, **25**(21):9478-9490.

10. Ptacek J, Devgan G, Michaud G, Zhu H, Zhu X, Fasolo J, Guo H, Jona G, Breitkreutz A, Sopko R, McCartney RR, Schmidt MC, Rachidi N, Lee SJ, Mah AS, Meng L, Stark MJ, Stern DF, De Virgilio C, Tyers M, Andrews B, Gerstein M, Schweitzer B, Predki PF, Snyder M: **Global analysis of protein phosphorylation in yeast**. *Nature* 2005, **438**(7068):679-684.

11. Su SS, Mitchell AP: **Identification of functionally related genes that stimulate early meiotic gene expression in yeast**. *Genetics* 1993, **133**(1):67-77.

12. Eaton S: **Retromer retrieves wntless**. *Dev Cell* 2008, **14**(1):4-6.

13. McLysaght A, Baldi PF, Gaut BS: **Extensive gene gain associated with adaptive evolution of poxviruses**. *Proceedings of the National Academy of Sciences of the United States of America* 2003, **100**(26):15655-15660.

14. Tulman ER, Afonso CL, Lu Z, Zsak L, Kutish GF, Rock DL: **The genome of canarypox virus**. *J Virol* 2004, **78**(1):353-366.

15. McFadden G: **Poxvirus tropism**. *Nat Rev Microbiol* 2005, **3**(3):201-213.

16. Husain M, Moss B: **Role of receptor-mediated endocytosis in the formation of vaccinia virus extracellular enveloped particles**. *J Virol* 2005, **79**(7):4080-4089.
