# Supplementary material for: On the origins of arrestin and rhodopsin
Source: BMC Evol Biol. 2008 Jul 29;8:222. doi: 10.1186/1471-2148-8-222 (PMC2515105; doi:10.1186/1471-2148-8-222)
Supplement: Additional file 2 — Supplementary methods. A Doc file with the methods used in the discovery and analysis of protist arrestins, discovery and protein sequence alignment of zebrafish and human arrestins, secondary and tertiary structure predictions of alpha arrestins, and phylogenetic analysis of canarypox virus arrestin. All protein sequence annotations are also provided here. [file 1471-2148-8-222-S2.doc]

**Additional file 2. Methods**

**Protist Arrestins**

Position-Specific Iterated (PSI) BLAST [1] was used to find all alpha arrestins in the GenBank protein database. PSI-BLAST uses multiple search iterations to “learn” a pattern from related sequences (i.e., building a score matrix after each round). All protist arrestins were scanned for known protein patterns using the Pfam Hidden Markov Modeling server HMMER [2, 3] and the Pfam database [4, 5] [see Additional file 3]. We updated new sequences for this submission by using the Pfam database of HMMER-identified arrestins. Those added from Pfam are the last three listed *T.cruzi* and all the *T. thermophila* proteins. The HMMER confidence E scores of all proteins were re-determined with the updated Pfam matrix. Notably, HMMER scores must be interpreted with the caveat that the Pfam pattern matrix is made from the conclusive subset of proteins that are derived from a very select subset of organisms (i.e., model organisms that are sequenced and annotated). On the other hand, the PSI-BLAST detection (above) was based on creating a matrix de novo with all available sequences. Thus we expected PSI-BLAST to pick up some false negatives (or low confidence) from HMMER. To further test the three proteins with the weakest HMMER scores, we conducted BLASTP analysis (to identify the most similar proteins by pair-wise comparison). The putative arrestin region was used to query all proteins in GenBank [Additional file 3]. The results suggest that all members (with one possible exception) in Additional file 3 are true arrestins. Importantly, the two proteins with the lowest confidence scores (*Entamoeba* XP_654239.1 and *Tetrahymena* Q22QJ9) are supported by the presence of both Arrestin N and C domains.

**Alignment of Zebrafish and Human Arrestins**

We used BLASTP and TBLASTN (NCBI) to search for all zebrafish, Takifugu and Tetraodon arrestins in GenBank. We found the largest number of conclusive hits in zebrafish. However, the zebrafish genome annotation is not sufficiently complete to identify all arrestins. We identified all cloned and predicted zebrafish arrestins, and were able to confidently assemble two other arrestins using ESTs and genome sequence (Arrb1 and Arrdc3A, [see Additional file 2]). All the human arrestins are now well annotated in diverse public databases. Mammalian Arrdc2 has alternative N-terminal sequences; we only included one isoform. We excluded all fish sequences that appeared to be generated by alternative-splicing or by a very recent gene duplication (or sequencing errors) and those with major sections of conserved sequence missing (i.e., dramatically divergent or artifactual). Clustal W was used to perform the multiple sequence alignments shown in Additional file 4 (using default settings, SDSC Biology Workbench [6]). Few manual corrections were made. Key regions and residues of the Arr3 and Arrb1 crystal structures are mapped on the aligned ARR3 [Additional file 4] and ARRB1 [Additional file 6] sequences [7, 8].

**Structure Predictions**

The secondary structure prediction for Additional file 4 was done with the program Protein Homology/analogY Recognition Engine (Phyre) [9], the successor of 3D-PSSM [10]. The secondary structure predictions for Additional file 6 were done with PELE (SDSC Biology Workbench [6]), which implements eight commonly used algorithms in concert. The 3D structure predictions mentioned with confidence scores in the article text were predicted using GenThreader [11], implemented on the PSIPRED Protein Structure Prediction Server [12, 13]. Similar 3D prediction results were generated by the Phyre program.

**Phylogenetic analysis of canarypox virus arrestin**

In order to maximize the information content for tree building we first identified the closest relatives of canarypox virus (CNPV) arrestin. Examination of multiple alignments and phylogenetic trees unambiguously narrowed the closest relatives in GenBank to the sequences shown in Additional file 6. Sequences were aligned by Clustal W (SDSC Biology Workbench [6]). A neighbor-joining tree was made from amino acid pairwise distance, with Poisson correction (MEGA2; [14]). Bootstrap values shown are from 500 repetitions. The same tree topology was obtained using the Maximum parsimony tree building method.

**Conceptual translation of DNA sequence assemblies (with DNA Accession Numbers)**

>Arrb1_BRARE (assembly of BC083452.1, EB942633.1 and UCSC Danio Rerio Mar. 2006 Genescan exon chr15:56,786,948-56,787,015)

MGDKGTRVFKKASPNGKLTVYLGKRDFVDHVDLVEPVDGVVLIDPEYLKERKVFVTLTCAFRYGREDLDV

LGLTFRKDLFVANIQAFPPVPEEKKSLTRLQERLIKKLGEHAYPFTFEIPPNLPCSVTLQPGPEDTGKAC

GVDFEVKAFCAENVEEKIHKRNSVRLVIRKVQYAPEKPGPQPMAETTRQFLMSDKPLHLEASLDKEIYYH

GEPISVNVHVTNNTNKTVKKIKISVRQYADICLFNTAQYKCPVAIEESDDIVAPSATFCKVYTLTPFLAN

NREKRGLALDGKLKHEDTNLASSTLLREGANKEILGIIVSYKVKVKLVVSRGGLLGDLASSDVAVELPFT

LMHPKPLEESIYRDAPENDAPIDTNLIEFDTNDDDIIFEDFARQRLIGAK

>Arrdc_chick XP_424699.1 plus 12aa term (CD738677.1, AADN02047353.1)
MVLGKVKSLTISFDCLNDSNVPVYSSGDTVSGRVNLEVTGEIRVKSLKIHARGHAKVRWTESRNAGSNTA
YTQNYTEEVEYFNHKDVLIGHERDDDNSEEGLHTIPSGRHEYAFSFELPQTPLATSFEGRHGSVRYWVKA
ELHRPWLLPVKLKKEFTVFEHIDINTPSLLSPQAGTKEKTLCCWFCTSGPISLSAKIERKGYTPGESIQI
FAEIENCSSRVVVPKAAIYQTQAFYAKGKMKEVKQLVANLRGESLSSGKTETWNGKVLKIPPVSPSILDC
SIIRVEYSLMVYVDIPGAMDLFLNLPLVIGTIPLHPFGSRTSSVSSQCSMNMNWLGLTLPERPEAPPSYA
EVVTEEQRQSSLPPIAACDDFERALQGPLFAYIQEFRFLPPPLYSEIDPNPDQPTDDRPSCPSR

>Arrdc3A_BRARE (assembly of EB952186.1, DN858546 and UCSC Danio Rerio Mar. 2006 chr5:58,318,748-58,319,214)

MVLGKVKSFIVSYDCLNDSNVPVFSSGDSVSGRVIIEVTGEIRVKSLKINAKGFAKVRWTESRNAGSSTA

YTQNYTEEVEYLNHRDILIGHERDDDNSEEGLTTIHSGRHEYAFSFELPQTPLATSFEGKHGSVRYWVKA

ELHRPWLLPMKTKKEFTVFEHIDINTPLLLSPQAGTKEKTLCCWFCTSGPISLSAKIERKGYTPGESIQI

FAEIENCSSRMVVPKAAIYQTQTFFAKGKMKEIKQLVANIRGESLSSGKTETWNGKMLKIPPVSPSILDC

SIIRVEYSLMVYVDIPGAMNLSLNLPLVIGTIPLHPFGSRTSSVSSQCSMTMSWLGMALPERPEAPPAYA

EVVTEEQRQNCLEVSPGRENYDGPLFAYIQEFRFRPPPPYSEIDPHPDQATSTAEQRLDTCPSR

>Arrb_HYDMA (assembly of CV465032.1, DN816101.2, DT612736.1, CO371080.1, DT617742.1, CV464427.1, CV464909.1)

MEKEKSSKDSSPVKTKVLKENTAISEKKGGTRVFKKSTPNGKITVYIGKRDFYDHIEHCEPVDGVVLVDP

EHVKDKTVFCHVLAAFRYGREDLDVLGLTFRKDLFLAKKQVYPPEPVENKPEELTRLQERLIKKLGKNAF

PFKFQLPSSAPSSVTLQPAPGGVDYELRVYMSETADEKEYKRNSVRLAIRKITYAPPVIAAQPTVEQHKE

FMMCPHPLILEASLDKGMYYHGESIAVNVHISNRSNKTVKKIKITVRQYADICLFSTAQYKCPVASIESE

EGFPVGQSGTLSKIILLTPLLANNKDKPWLGLDGQLKHEDTNLASSTIMDQNTPKENLGIIVHYKVKIRL

HVAYGGDLSVELPFVLTHPKPPETSPSTTPVLARKETTVPGEEENPKIENDLINFDTDGSVKHPDDELVF

EEFARMRVRGEHYGDSNA

>Opsin_HYDMA (assembly of CB073527.1, CN554795.1, CN554455.1, CB271253.1; confirmed by many genomic trace sequences)

MAFVFIIVFLSFLCGFSVILNVTVVLTILAKGNTKNTRDVILMSLAICDGVQCTIGYPVELFGYANYKNP

SLSEKFCKPSGFIVMYLALTAIAHLVCLCIYRYLTIVYPLKLQIFLTKSNWSACGCIAFCWIYGLFWSLS

PLLGWNEIVREKQDTYRCSINLYPDNEIKSSYLYALAIFCYLIPLIIIIYCSLKVRSELRNMLKMCKQIS

GVEANITKVTYRIEKQDFISVSFIIASFFTVWTPYAVCVFYLTIGKKLPPSFLTYCALFAKSSTILNPII

YCLMYKKFRQTLQSKFGKLFNNPTVTPAV

**Taxonomy abbreviations**

BRARE, *Danio rerio* (zebrafish; Vertebrata); CIOIN, *Ciona intestinalis* (Urochordata); DROME, *Drosophila m*. (Arthropoda); CAEEL, *C. elegans* (Nematoda); HYDMA, *Hydra magnipapillata* (Cnidaria), PARTE, *Paramecium tetraurelia* (Alveolata); EMENI, *Emericella nidulans* (Fungi/Ascomycota); SCHPO, *Schizosaccharomyces pombe* (Fungi/Ascomycota); 9STRA, stramenopiles; DICDI, *Dictyostelium discoideum*  (Protista, Mycetozoa)*.*

**Protein Sequence Annotations**

Q8LKK8_9STRA (str_protist_Q8L), Q6BGG1_PARTE (par_protist_Q6B), Q86KB1_DICDI (sl_mold_Q86KB1), Q55CH0_DICDI (sl_mold_Q55CH0), Q54HT7_DICDI (sl_mold_Q54HT7), Q86KB1_DICDI (sl_mold_Q54CH1), YA4C_SCHPO_Q09729 (fis_yeast_YA4C), YC9F_SCHPO_Q09889 (fis_yeast_YC9F), SPBC2D10.04_SCHPO_O74798 (fis_yeast_SPBC2), CreD_EMENI_Q6SIF1 (asc_yeast_CreD), PALF_EMENI_P78612 (asc_fungus_PALF), ARRB_HYDMA assembly below (hydra_ARRB), Y17G7B.11_CAEEL_Q9XXI4 (worm_Y17G7B.11), F48F7.7_CAEEL_Q20577 (worm_F48F7.7), Y17G7B.14_CAEEL_Q9XXI0 (worm_Y17G7B.14), Q9GYT4_CAEEL (worm_Q9GYT4), 471R06B9.3_CAEEL_O17982 (worm_R06B9.3), R06B9.1_CAEEL_O17981 (worm_R06B9.1), Q9GYT5_CAEEL (worm_Q9GYT5), T20D4.6_CAEEL_P91469 (worm_T20D4.6), ARRB_CAEEL_P51485 (worm_ARRB), Y51B9A.4_CAEEL_Q9XXC4 (worm_Y51B9A.4), R06B9.2_CAEEL_O17984 (worm_R06B9.2), ZK938.4_CAEEL_Q23674 (worm_ZK938.4), Y49E10.24_CAEEL_Q9XTU0 (worm_Y49E10.24), T12D8.4_CAEEL_O45782 (worm_T12D8.4), R06B9.4_CAEEL_O17983 (worm_R06B9.4), F15A4.9_CAEEL_O17812 (worm_F15A4.9), F40F8.8_CAEEL_Q20232 (worm_F40F8.8), C04C11.2_CAEEL_Q17624 (worm_C04C11.2), CG18745-PA_DROME_Q9I7L0 (fly_CG18745-PA), CG18746-PA_DROME_Q9I7K8 (fly_CG18746-PA), CG18747-PA_DROME_Q9I7L1 (fly_CG18747-PA), CG2641-PA_DROME_Q9VI00 (fly_CG2641-PA), CG3014-PA_DROME_Q9VHZ9 (fly_CG3014-PA), CG1487-PA_DROME_Q9V393 (fly_CG1487-PA), ARRA_DROME_P15372 (fly_ARRA), ARRB_DROME_P19107 (fly_ARRB), CG32683-PA_DROME_Q9W2V2 (fly_RH70434p), RE33762p_DROME_Q8SYU5 (fly_RE33762p), CG1105-PA_DROME_Q9VI53 (fly_CG1105-PA), CG2993-PA_DROME_Q9VHZ7 (fly_CG2993-PA), CG14696-PA_DROME_Q9VGU7 (fly_CG14696-PA), CG7047-PB_DROME_Q9W0U5 (fly_CG7047-PB), CG18744-PA_DROME_Q9I7K7 (fly_CG18744-PA), CG4674-PA_DROME_Q9VGU4 (fly_CG4674-PA), CG18268-PA_DROME_Q9VHZ8 (fly_CG18268-PA), Arrestin_CIOIN_Q8MYB1 (protochord_ARR), Q6ZM71_BRARE (fish_Q6ZM71), TXNIP_BRARE_Q7ZWB6 (fish_TXNIP), Q6TEM6_BRARE (fish_Q6TEM6), Zgc-66353_BRARE_Q6PFK2 (fish_Zgc-66353), Zgc-92697_BRARE_Q6DH07 (fish_Zgc-92697), ARR3_BRARE_Q6PBM9 (fish_ARR3), Zgc-66109_BRARE_Q6NUV2 (fish_Zgc-66109), ARRB1_BRARE assembly below (fish_ARRB1), ARRDC3A_BRARE assembly below (fish_ARRDC3A), NP_001004605_ZF (fish_NP_0010046), XP_683827_BRARE (fish_XP_683827), ARRDC1_HUMAN_Q8N5I2 (human_ARRDC1), VDUP1_HUMAN_Q9H3M7 (human_TXNIP), ARRDC2_HUMAN_Q8TBH0 (human_ARRDC2), ARRDC3_HUMAN­_Q96B67 (human_ARRDC3), ARRDC4_HUMAN_Q8NCT1 (human_ARRDC4), ARRB2_HUMAN_Q6ICT3 (human_ARRB2), ARRB1_HUMAN_P49407 (human_ARRB1), ARR3_HUMAN_P36575 (human_ARR3), ARRS_HUMAN_P10523S (human_ARRS), ARRDC5_HUMAN_ XP_933564 (human_ARRDC5).

**References**
