# Supplementary material for: On the origins of arrestin and rhodopsin
Source: BMC Evol Biol. 2008 Jul 29;8:222. doi: 10.1186/1471-2148-8-222 (PMC2515105; doi:10.1186/1471-2148-8-222)
Supplement: Additional file 3 — Table of protist arrestins. A PDF file with Table 1. Details of protist arrestins. [file 1471-2148-8-222-S3.pdf]

Protist Phyla	Species	Accession Number (length, aa)	N Arrestin Domain Pos. (HMMER E score)	C Arrestin Domain Pos. (HMMER E score)	Other Domains (HMMER E score), Function; Notes
Mycetozoa, Dictyosteliida	*Dictyostelium discoideum*	Q86KB1 (617)	182-319 (0.0011)	337-465 (3.6e-09)	C2, 6-90 (6.9e-28), lipid/membrane binding; SAM_1, protein-protein homodimerizing/polymerizing 552-614 (0.0029)
		Q55CH0 (654)	178-326 (0.00025)	344-478 (0.00016)	C2, 6-86 (5.3e-23); SAM_1, 567-627 (0.015)
		Q54HT7 (1030)	*490-743 (1.9)*; 692-719 (0.00056)	826-1012 (0.00025)	
		Q54CH1 (580)	144-292 (1.2e-06)	313-441 (8.2e-05)	FYVE, 463-529 (2e-22), phosphatidylinositol-3-phosphate/membrane binding
Entamoebidae	*Entamoeba histolytica*	XP_656065.1 (395)	*(only detected by BLAST)**	*171-303 (0.43)**	FYVE, 328-394 (1.6e-22); *validated by BLAST (1-327 matches Q54CH1 above, E=6e-04)
		XP_653204.1 (615)	4-161 (1e-07)	*173-293 (0.063)*	WW, 337-362 (0.00096), protein-protein binding of Pro-Tyr and phospho-Ser/Thr motifs, associated with signal transduction; WW 383-411 (3.4e-08); C2, 430-512 (4.6e-06)
		XP_654239.1 (860)	5-135 (0.035)*	*149-274 (0.081)**	*suggested by BLAST (1-274 is most similar to mouse ARRDC2 in all GenBank, E=0.075)
		XP_656411.1 (611)	25-161 (0.0075)	178-299 (0.011)	*C2, 425-506 (0.97)*
		XP_657579.1 (1122)	6-148 (0.0033)	*173-289 (4.0)*	WW, 368-396 (0.021); *C2, 409-487 (0.47)*
Euglenozoa, Kinetoplastida	*Leishmania major*	CAJ09387.1 (809)	15-209 (0.0013) ; 148-172 (2.3e-05)	*517-657 (6.3)*	
		AAZ14274.1 (603)	5-220 (0.008); 148-170 (0.00032)		

	Trypanosoma cruzi	EAN93375.1 (594)	13-185 (0.00079)	*351-482 (0.15)*	
		Q4DDP4 (594)	13-185 (0.00097)	*351-482 (0.10)*	
		Q4E5Y2	9-176 (2.1e-05)		
		Q4DTF1 (515)	56-223 (3.5e-05)		
Stramenopiles	*Phytophthora sojae*	Q8LKK8/Q8LKK5 (426/416)	75-222 (7.9e-07)	242-377 (0.0021)	
Alveolata	*Paramecium tetraurelia*	Q6BGG1 (394)	12-167 (6.1e-09)	*186-328 (0.24)*	
	Tetrahymena thermophila	Q23Z84 (705)	199-359 (1.1e-06)	379-520 (0.018)	
		Q22QJ8 (2207)	94-252 (0.00018)	271-394 (0.029)	H-type lectin, 538-611 (1.4e-15), carbohydrate binding involved in self/non-self cell recognition
		Q23QU8 (421)	17-172 (0.0015)	*188-316 (0.22)*	
		Q23FQ6 (463)	17-170 (0.0076)	*188-316 (0.076)*	
		Q23I98 (404)	30-182 (0.00031)	202-328 (0.0015)	
		Q22BC1 (407)	18-171(8.2e-05)	*191-323 (0.099)*	
		Q23Z85 (472)	15-173 (5.1e-07)	190-331 (0.0015)	
		Q22F38 (333)	8-160 (3.7e-06)	*176-304 (0.23)*	
		Q22CF7 (541)	17-150 (4.7e-05)	179-329 (0.042)	
		Q23JY6 (3032)	19-177 (2.4e-05)	*200-331 (0.2)*; 200-225 (0.014)	cNMP_binding, 1157-1266 (4.1e-09), cyclic nucleotide-binding; PP2C, 2549-2840 (6e-56), Mn/Mg++ dependent protein serine/threonine phosphatase
		Q23JY5 (391)	18-178 (0.00019)	*195-325 (30.0)*	
		Q229Z0 (554)	17-169 (0.0015)	*188-324 (0.72)*	
		Q22CU7 (1194)	804-972 (0.0019)	*1005-1120 (2.4)*	
		Q229J7 (408)	17-172 (0.0019)	*187-307 (1.4)*	
		Q22QJ9 (2277)	1820-1972 (0.014)*	*1991-2119 (0.88)**	H-type lectin, 39-112 (4.2e-13); *validated by BLAST (1820-2119 matches Q6BGG1 above, E=3e-05)
