# Supplementary material for: On the origins of arrestin and rhodopsin
Source: BMC Evol Biol. 2008 Jul 29;8:222. doi: 10.1186/1471-2148-8-222 (PMC2515105; doi:10.1186/1471-2148-8-222)
Supplement: Additional file 4 — Multiple sequence alignment of human and zebrafish arrestins. A PDF file showing a multiple sequence alignment of the human arrestin family and select fish arrestins. It shows protein conservation and divergence of different vertebrate arrestins. Alignment region of beta arrestin helix I suggests this structural element is absent in alpha arrestins. The conservation of PY motifs in alpha arrestins suggests they are functional. [file 1471-2148-8-222-S4.pdf]

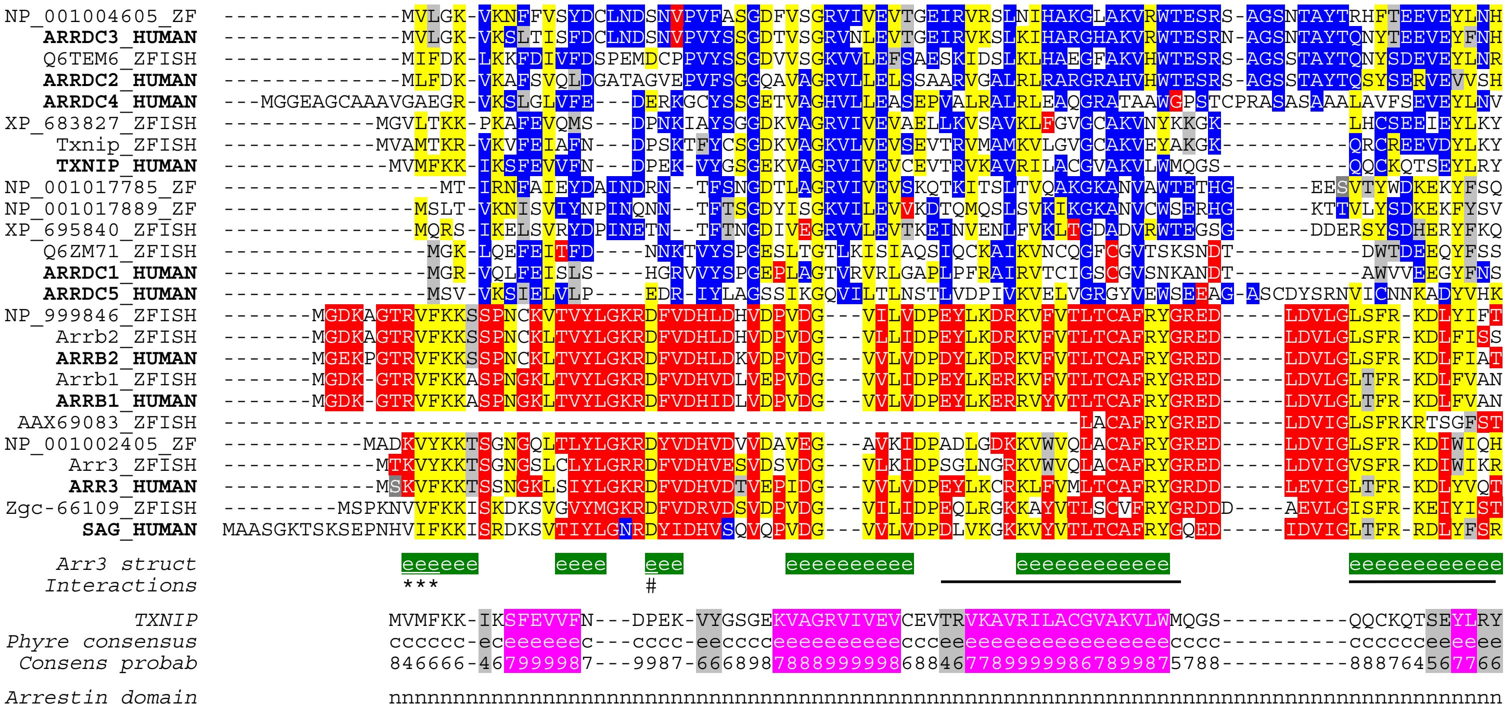
NP_001004605_ZF --------------MVLGK-VKNFFVSYDCLNDSNVPVFASGDFVSGRVIVEVTGEIRVRSLNIHAKGLAKVRWTESRS-AGSNTAYTRHFTEEVEYLNH
ARRDC3_HUMAN --------------MVLGK-VKSLTISFDCLNDSNVPVYSSGDTVSGRVNLEVTGEIRVKSLKIHARGHAKVRWTESRN-AGSNTAYTQNYTEEVEYFNH
Q6TEM6_ZFISH --------------MIFDK-LKKFDIVFDSPEMDCPPVYSSGDVVSGKVVLEFSAESKIDSLKLHAEGFAKVHWTESRS-AGSSTAYTQNYSDEVEYLNR
ARRDC2_HUMAN --------------MLFDK-VKAFSVQLDGATAGVEPVFSGGQAVAGRVLLELSSAARVGALRLRARGRAHVHWTESRS-AGSSTAYTQSYSERVEVVSH
ARRDC4_HUMAN ---MGGEAGCAAAVGAEGR-VKSLGLVFE---DERKGCYSSGETVAGHVLLEASEPVALRALRLEAQGRATAAWGPSTCPRASASAAALAVFSEVEYLNV
XP_683827_ZFISH ------------MGVLTKK-PKAFEVQMS---DPNKIAYSGGDKVAGRVIVEVAELLKVSAVKLFGVGCAKVNYKKGK----------LHCSEEIEYLKY
Txnip_ZFISH ------------MVAMTKR-VKVFEIAFN---DPSKTFYCSGDKVAGKVLVEVSEVTRVMAMKVLGVGCAKVEYAKGK----------QRCREEVDYLKY
TXNIP_HUMAN -------------MVMFKK-IKSFEVVFN---DPEK-VYGSGEKVAGRVIVEVCEVTRVKAVRILACGVAKVLWMQGS----------QQCKQTSEYLRY
NP_001017785_ZF -----------------MT-IRNFAIEYDAINDRN--TFSNGDTLAGRVIVEVSKQTKITSLTVQAKGKANVAWTETHG------EESVTYWDKEKYFSQ
NP_001017889_ZF ---------------MSLT-VKNISVIYNPINQNN--TFTSGDYISGKVILEVVKDTQMQSLSVKIKGKANVCWSERHG------KTTVLYSDKEKFYSV
XP_695840_ZFISH ---------------MQRS-IKELSVRYDPINETN--TFTNGDIVEGRVVLEVTKEINVENLFVKLTGDADVRWTEGSG------DDERSYSDHERYFKQ
Q6ZM71_ZFISH ----------------MGK-LQEFEITFD----NNKTVYSPGESLTGTLKISIAQSIQCKAIKVNCQGFCGVTSKSNDT----------DWTDEEQYFSS
ARRDC1_HUMAN ----------------MGR-VQLFEISLS----HGRVVYSPGEPLAGTVRVRLGAPLPFRAIRVTCIGSCGVSNKANDT----------AWVVEEGYFNS
ARRDC5_HUMAN ----------------MSV-VKSIELVLP----EDR-IYLAGSSIKGQVILTLNSTLVDPIVKVELVGRGYVEWSEEAG-ASCDYSRNVICNNKADYVHK
NP_999846_ZFISH -------MGDKAGTRVFKKSSPNCKVTVYLGKRDFVDHLDHVDPVDG---VILVDPEYLKDRKVFVTLTCAFRYGRED-----LDVLGLSFR-KDLYIFT
Arrb2_ZFISH -------MGDKAGTRVFKKSSPNCKLTVYLGKRDFVDHLDHVDPVDG---VLLIDPEYLKDRKVFVTLTCAFRYGRED-----LDVLGLSFR-KDLFISS
ARRB2_HUMAN -------MGEKPGTRVFKKSSPNCKLTVYLGKRDFVDHLDKVDPVDG---VVLVDPDYLKDRKVFVTLTCAFRYGRED-----LDVLGLSFR-KDLFIAT
Arrb1_ZFISH -------MGDK-GTRVFKKASPNGKLTVYLGKRDFVDHVDLVEPVDG---VVLIDPEYLKERKVFVTLTCAFRYGRED-----LDVLGLTFR-KDLFVAN
ARRB1_HUMAN -------MGDK-GTRVFKKASPNGKLTVYLGKRDFVDHIDLVDPVDG---VVLVDPEYLKERRVYVTLTCAFRYGRED-----LDVLGLTFR-KDLFVAN
AAX69083_ZFISH ---LACAFRYGRED----LDVIGLSFRKRTSGFST
NP_001002405_ZF ------------MADKVYKKTSGNGQLTLYLGKRDYVDHVDVVDAVEG---AVKIDPADLGDKKVWVQLACAFRYGRED-----LDVIGLSFR-KDIWIQH
Arr3_ZFISH -------------MTKVYKKTSGNGSLCLYLGRRDFVDHVESVDSVDG---VLKIDPSGLNGRKVWVQLACAFRYGRED-----LDVIGVSFR-KDIWIKR
ARR3_HUMAN -------------MSKVFKKTSSNGKLSIYLGKRDFVDHVDTVEPIDG---VVLVDPEYLKCRKLFVMLTCAFRYGRDD-----LEVIGLTFR-KDLYVQT
Zgc-66109_ZFISH ---------MSPKNVVFKKISKDKSVGVYMGKRDFVDRVDSVDPVDG---VILIDPEQLRGKKAYVTLSCVFRYGRDDD---AEVLGISFR-KEIYIST
SAG_HUMAN MAASGKTSKSEPNHVIFKKISRDKSVTIYLGNRDYIDHVSQVQPVDG---VVLVDPDLVKGKKVYVTLTCAFRYGQED-----IDVIGLTFR-RDLYFSR
Arr3 struct eeeeee eeee eee eeeeeeeeee eeeeeeeeeeee eeeeeeeeeeee
Interactions *** #
TXNIP MVMFKK-IKSFEVVFN---DPEK-VYGSGEKVAGRVIVEVCEVTRVKAVRILACGVAKVLWMQGS----------QQCKQTSEYLRY
Phyre consensus cccccc-eceeeeeeec---cccc-eeccccceeeeeeeeeecccceeeeeeeeeeeeeeeeecccc----------cccccceeeeee
Consens probab 846666-4679999987---9987-66689878889999999868846778999998678998 75788----------888764567766
Arrestin domain nnnnnnnnnnnnnnnnnnnnnnnnnnnnnnnnnnnnnnnnnnnnnnnnnnnnnnnnnnnnnnnnnnnnnnnnnnnnnnnnnnnnnnnnn

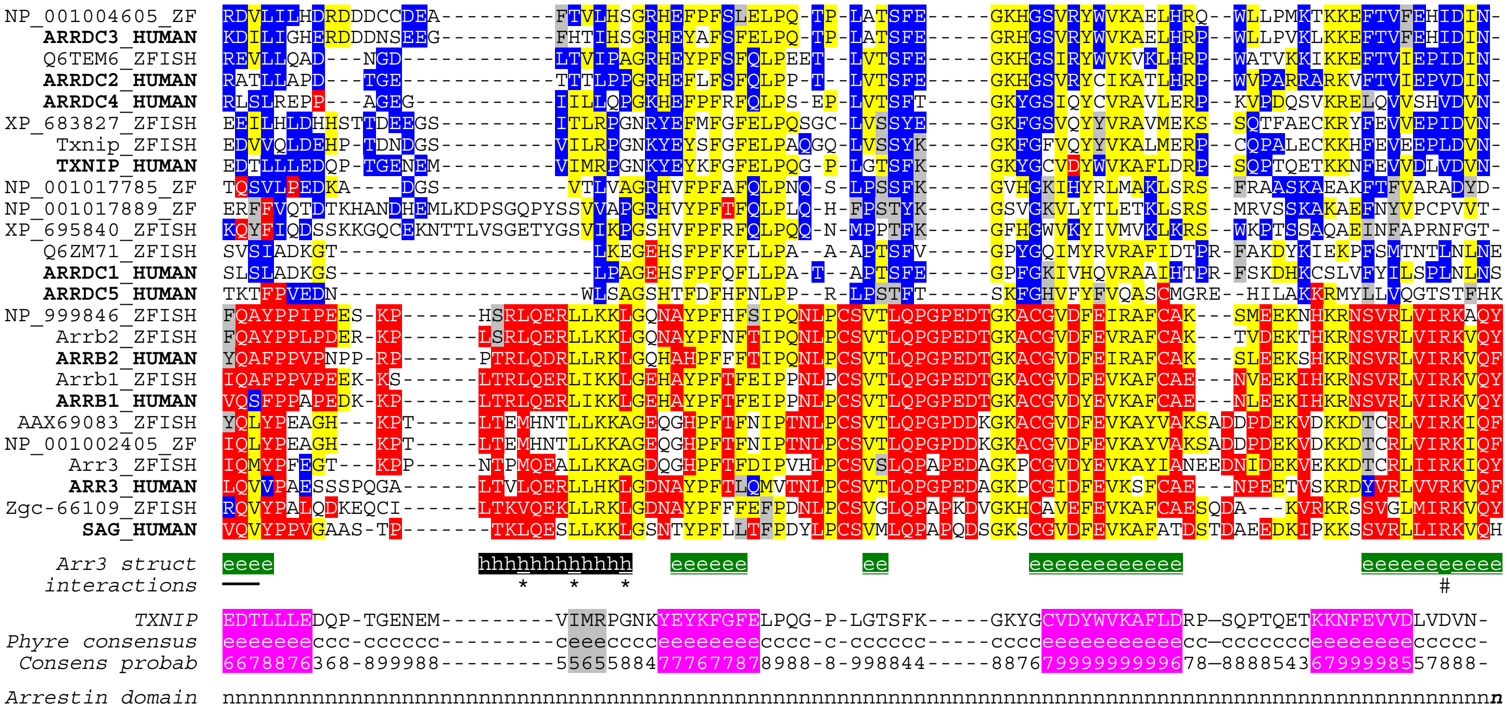
NP_001004605_ZF RDVLILHDRDDDCCDEA---------FTVLHSGRHEFPFSLELPQ-TP-LATSFE-----GKHGSVRYWVKAELHRQ--WLLPMKTKKEFTVFEHIDIN-
ARRDC3_HUMAN KDILIGHERDDDNSEEG---------FHTIHSGRHEYAFSFELPQ-TP-LATSFE-----GRHGSVRYWVKAELHRP--WLLPVKLKKEFTVFEHIDIN-
Q6TEM6_ZFISH REVLLQAD---NGD------------LTVIPAGRHEYPFSFQLPEET--LVTSFE-----GKHGSIRYWVKVKLHRP--WATVKKIKKEFTVIEPIDIN-
ARRDC2_HUMAN RATLLAPD---TGE------------TTTLPPGRHEFLFSFQLPP-T--LVTSFE-----GKHGSVRYCIKATLHRP--WVPARRARKVFTVIEPVDIN-
ARRDC4_HUMAN RLSLREPP---AGEG-----------IILLQPGKHEFPFRFQLPS-EP-LVTSFT-----GKYGSIQYCVRAVLERP--KVPDQSVKRELQVVSHVDVN-
XP_683827_ZFISH EEILHLDHHSTTDEEGS---------ITLRPGNRYEFMFGFELPQSGC-LVSSYE-----GKFGSVQYYVRAVMEKS--SQTFAECKRYFEVVEPIDVN-
Txnip_ZFISH EDVVQLDEHP-TDNDGS---------VILRPGNKYEYSFGFELPAQGQ-LVSSYK-----GKFGFVQYYVKALMERP--CQPALECKKHFEVEEPLDVN-
TXNIP_HUMAN EDTLLLEDQP-TGENEM---------VIMRPGNKYEYKFGFELPQG-P-LGTSFK-----GKYGCVDYWVKAFLDRP--SQPTQETKKNFEVVDLVDVN-
NP_001017785_ZF TQSVLPEDKA---DGS----------VTLVAGRHVFPFAFQLPNQ-S-LPSSFK-----GVHGKIHYRLMAKLSRS--FRAASKAEAKFTFVARADYD-
NP_001017889_ZF ERFFVQTDTKHANDHEMLKDPSGQPYSSVVAPGRHVYPFTFQLPLQ-H-FPSTYK-----GSVGKVLYTLETKLSRS--MRVSSKAKAEFNYVPCPVVT-
XP_695840_ZFISH KQYFIQDSSKKGQCEKNTTLVSGETYGSVIKPGSHVFPFRFQLPQQ-N-MPPTFK-----GFHGWVKYIVMVKLKRS--WKPTSSAQAEINFAPRNFGT-
Q6ZM71_ZFISH SVSIADKGT--------------------LKEGEHSFPFKFLLPA-A-APTSFV-----GPYGQIMYRVRAFIDTPR-FAKDYKIEKPFSMTNTLNLNE
ARRDC1_HUMAN SLSLADKGS--------------------LPAGEHSFPFQFLLPA-T--APTSFE-----GPFGKIVHQVRAAIHTPR-FSKDHKCSLVFYILSPLNLNS
ARRDC5_HUMAN TKTFPVEDN-------------------WLSAGSHTFDFHFNLPP--R-LPSTFT-----SKFGHVFYFVQASCMGRE--HILAKKRMYLLVQGTSTFHK
NP_999846_ZFISH FQAYPPIPEES-KP------HSRLQERLLKKLGQNAYPFHFSIPQNLPCSVTLQPGPEDTGKACGVDFEIRAFCAK---SMEEKNHKRNSVRLVIRKAQY
Arrb2_ZFISH FQAYPPLPDER-KP------LSRLQERLLKKLGQNAYPFNFTIPQNLPCSVTLQPGPEDTGKACGVDFEVRAFCAK---TVDEKTHKRNSVRLVIRKVQY
ARRB2_HUMAN YQAFPPVPNPP-RP------PTRLQDRLLRKLGQHAHPFFFTIPQNLPCSVTLQPGPEDTGKACGVDFEIRAFCAK---SLEEKSHKRNSVRLVIRKVQF
Arrb1_ZFISH IQAFPPVPEEK-KS------LTRLQERLIKKLGEHAYPFTFEIPPNLPCSVTLQPGPEDTGKACGVDFEVKAFCAE---NVEEKIHKRNSVRLVIRKVQY
ARRB1_HUMAN VQSFPPAPEDK-KP------LTRLQERLIKKLGEHAYPFTFEIPPNLPCSVTLQPGPEDTGKACGVDYEVKAFCAE---NLEEKIHKRNSVRLVIRKVQY
AAX69083_ZFISH YQLYPEAGH---KPT-----LTEMHNTLLKKAGEQGHPFTFNIPTNLPCSVTLQPGPDDKGKACGVDFEVKAYVAKSADDPDEKVDKKDTCRLVIRKIQF
NP_001002405_ZF IQLYPEAGH---KPT-----LTEMHNTLLKKAGEQGHPFTFNIPTNLPCSVTLQPGPDDKGKACGVDFEVKAYVAKSADDPDEKVDKKDTCRLVIRKIQF
Arr3_ZFISH IQMYPFEGT---KPP-----NTPMQEALLKKAGDQGHPFTFDIPVHLPCSVSLQPAPEDAGKPCGVDYEVKAYIANEEDNIDEKVEKKDTCRLIIRKIQY
ARR3_HUMAN LQVVPAESSSPQGA------LTVLQERLLHKLGDNAYPFTLQMVTNLPCSVTLQPGPEDAGKPCGIDFEVKSFCAE---NPEETVSKRDYVRLVVRKVQF
Zgc-66109_ZFISH RQVYPALQDKEQCI------LTKVQEKLLRKLGDNAYPFFFEFPDNLPCSVGLQPAPKDVGKHCAVEFEVKAFCAESQDA---KVRKRSSVGLMIRKVQY
SAG_HUMAN VQVYPPVGAAS-TP-------TKLQESLLKKLGSNTYPFLLTFPDYLPCSVMLQPAPQDSGKSCGVDFEVKAFATDSTDAEEDKIPKKSSVRLLIRKVQH
Arr3 struct eeee hhhhhhhhhhhh eeeeeee ee eeeeeeeeeeee eeeeeeeeeeee
interactions * * * #
TXNIP EDTLLLEDQP-TGENEM---------VIMRPGNKYEYKFGFELPQG-P-LGTSFK-----GKYGCVDYWVKAFLDRP—SQPTQETKKNFEVVDLVDVN-
Phyre consensus eeeeeeeccc-cccccc---------ceeecccceeeeeeeecccc-c-cccccc-----cccceeeeeeeeeeeecc—cccccccceeeeeeeeccccc-
Consens probab 6678876368-899988---------556558847776778788988-8-998844-----8876799999999996 78—8888543679999855 7888-
Arrestin domain nnnnnnnnnnnnnnnnnnnnnnnnnnnnnnnnnnnnnnnnnnnnnnnnnnnnnnnnnnnnnnnnnnnnnnnnnnnnnnnnnnnnnnnnnnnnnnnnn

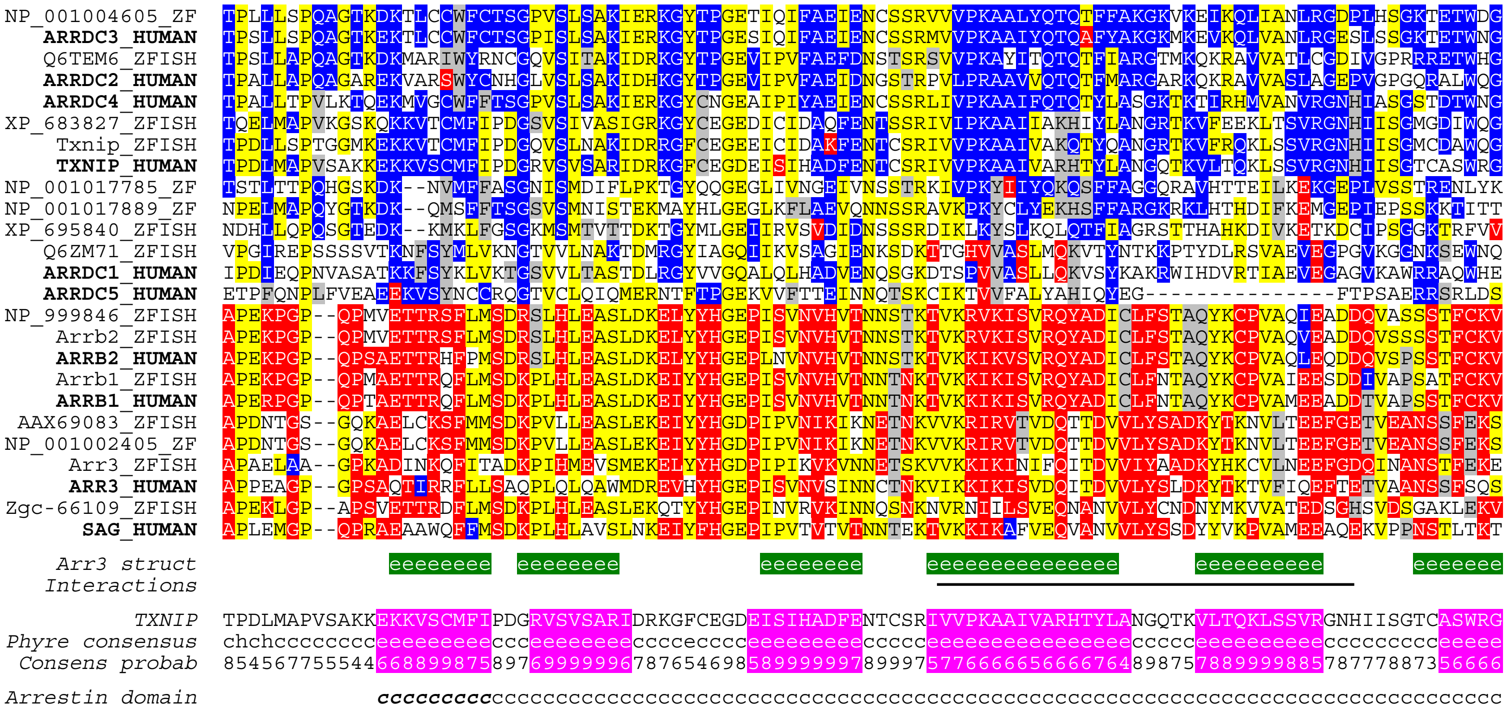
NP_001004605_ZF
ARRDC3_HUMAN
Q6TEM6_ZFISH
ARRDC2_HUMAN
ARRDC4_HUMAN
XP_683827_ZFISH
Txnip_ZFISH
TXNIP_HUMAN
NP_001017785_ZF
NP_001017889_ZF
XP_695840_ZFISH
Q6ZM71_ZFISH
ARRDC1_HUMAN
ARRDC5_HUMAN
NP_999846_ZFISH
Arrb2_ZFISH
ARRB2_HUMAN
Arrb1_ZFISH
ARRB1_HUMAN
AAX69083_ZFISH
NP_001002405_ZF
Arr3_ZFISH
ARR3_HUMAN
Zgc-66109_ZFISH
SAG_HUMAN
Arr3 struct
Interactions
TXNIP
Phyre consensus
Consens probab
Arrestin domain

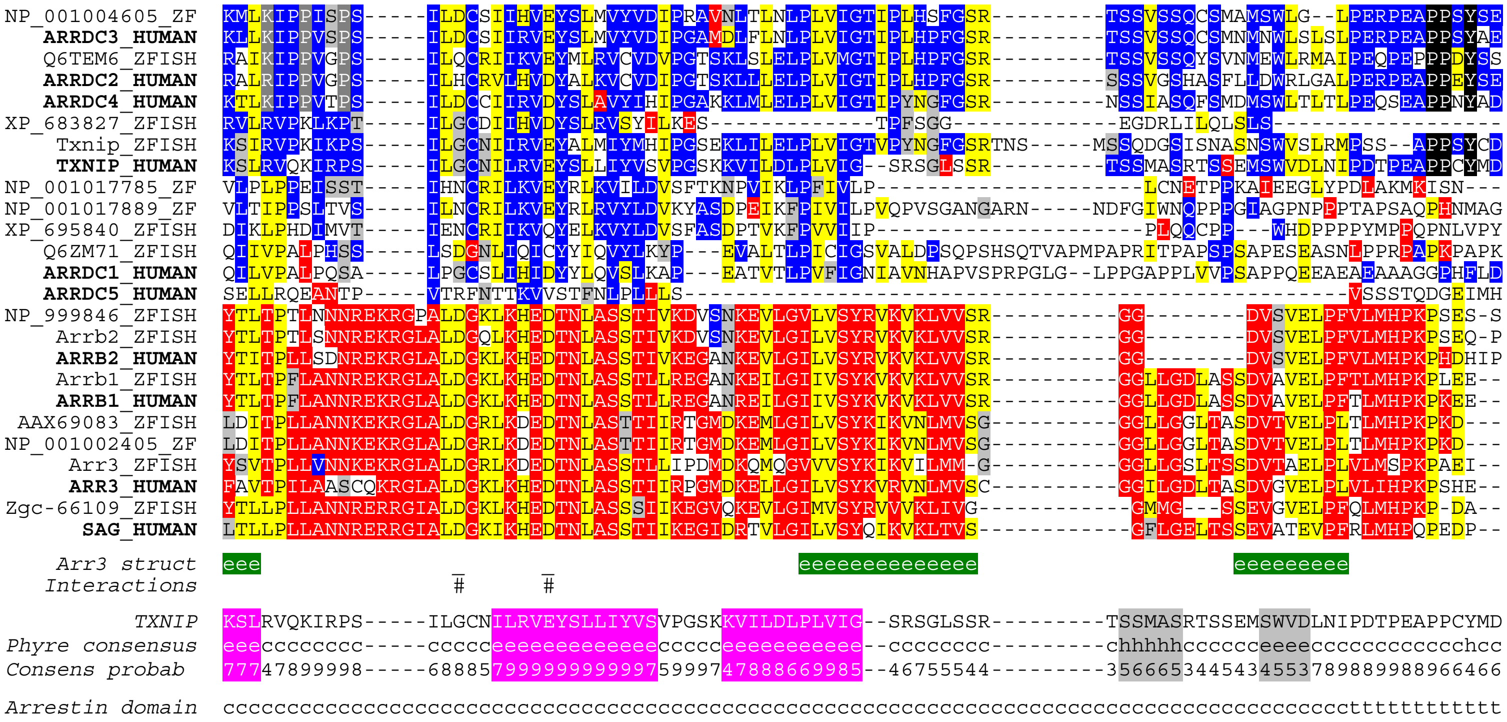
NP_001004605_ZF KMLKIPPISPS-----ILDCSIIHVEYSLMVYVDIPRAVNLTLNLPLVIGTIPLHSFGSR---------TSSVSSQCSMAMSWLG--LPERPEAPPSYSE
ARRDC3_HUMAN KLLKIPPVSPS-----ILDCSIIRVEYSLMVYVDIPGAMDLFLNLPLVIGTIPLHPFGSR---------TSSVSSQCSMNMNWLSLSLPERPEAPPSYAE
Q6TEM6_ZFISH RAIKIPPVGPS-----ILQCRIIKVEYMLRVCVDVPGTSKLSLELPLVMGTIPLHPFGSR---------TSSVSSQYSVNMEWLRMAIPEQPEPPDYSS
ARRDC2_HUMAN RALRIPPVGPS-----ILHCRVLHVDYALKVCVDIPGTSKLLLELPLVIGTIPLHPFGSR---------SSSVGSHASFLLDWRLGALPERPEAPPEYSE
ARRDC4_HUMAN KTLKIPPVTPS-----ILDCCIIRVDYSLAVYIHIPGAKKLMLELPLVIGTIPYNGFGSR---------NSSIASQFSMDMSWLTLTLPEQSEAPPNYAD
XP_683827_ZFISH RVLRVPKLKPT-----ILGCDIIHVDYSLRVSYILKES------------TPFSGG--------------EGDRLILQLSLS-----------------
Txnip_ZFISH KSIRVPKIKPS-----ILGCNIIRVEYALMIYMHIPGSEKLILELPLVIGTVPYNGFGSRTNS-----MSSQDGSISNASNSWVSLRMPSS--APPSYCD
TXNIP_HUMAN KSLRVQKIRPS-----ILGCNILRVEYSLLIYVSVPGSKKVILDLPLVIG--SRSGLSSR---------TSSMASRTSSEMSWVDLNIPDTPEAPPCYMD
NP_001017785_ZF VLPLPPEISST-----IHNCRILKVEYRLKVILDVSFTKNPVIKLPFIVLP--------------------LCNETPPKAIEEGLYPDLAKMKISN---
NP_001017889_ZF VLTIPPSLTVS-----ILNCRILKVEYRLRVYLDVKYASDPEIKFPIVILPVQPVSGANGARN----NDFGIWNQPPPGIAGPNPPPTAPSAQPHNMAG
XP_695840_ZFISH DIKLPHDIMVT-----IENCRIIKVQYELKVYLDVSFASDPTVKFPVVIIP--------------------PLQQCPP---WHDPPPPYMPPQPNLVPY
Q6ZM71_ZFISH QIIVPALPHSS-----LSDGNLIQICYYIQVYLKYP---EVALTLPICIGSVALDPSQPSHSQTVAPMPAPRITPAPSPSAPESEASNLPPRPAPKPAPK
ARRDC1_HUMAN QILVPALPQSA-----LPGCSLIHIDYYLQVSLKAP---EATVTLPVFIGNIAVNHAPVSPRPGLG--LPPGAPPLVVPSAPPQEEAEAEAAAGGPHFLD
ARRDC5_HUMAN SELLRQEANTP-----VTRFNTTKVVSTFNLPLLLS---VSSSTQDGEIMH
NP_999846_ZFISH YTLTPTLNNNREKRGPALDGKLKHEDTNLASSTIVKDVSNKEVLGVLVSYRVKVKLVVSR---------GG--------DVSVELPFVLMHPKPSES-S
Arrb2_ZFISH YTLTPTLSNNREKRGLALDGQLKHEDTNLASSTIVKDVSNKEVLGILVSYRVKVKLVVSR---------GG--------DVSVELPFVLMHPKPSEQ-P
ARRB2_HUMAN YTITPLLSDNREKRGLALDGKLKHEDTNLASSTIVKEGANKEVLGILVSYRVKVKLVVSR---------GG--------DVSVELPFVLMHPKPHDHIP
Arrb1_ZFISH YTLTPFLANNREKRGLALDGKLKHEDTNLASSTLLREGANKEILGIIVSYKVKVKLVVSR---------GGLLGDLASSDVAVELPFTLMHPKPLEE--
ARRB1_HUMAN YTLTPFLANNREKRGLALDGKLKHEDTNLASSTLLREGANREILGIIVSYKVKVKLVVSR---------GGLLGDLASSDVAVELPFTLMHPKPKEE--
AAX69083_ZFISH LDITPLLANNKEKRGLALDGRLKDEDTNLASTTIIRTGMDKEMLGILVSYKIKVNLMVSG---------GGLLGGLTASDVTVELPLTLMHPKPKD---
NP_001002405_ZF LDITPLLANNKEKRGLALDGRLKDEDTNLASTTIIRTGMDKEMLGILVSYKIKVNLMVSG---------GGLLGGLTASDVTVELPLTLMHPKPKD---
Arr3_ZFISH YSVTPLLVNNKEKRGLALDGRLKDEDTNLASSTLLIPDMDKQMQGVVVSYKIKVILMM-G---------GGLLGSLTSSDVTAELPLVLMSPKPAEI--
ARR3_HUMAN FAVTPILAASCQKRGLALDGKLKHEDTNLASSTIIRPGMDKELLGILVSYKVRVNLMVSC---------GGILGDLTASDVGVELPLVLIHPKPSHE--
Zgc-66109_ZFISH YTLLPLLANNRERRGIALDGKLKHEDTNLASSSIIKEGVQKEVLGIMVSYRVVVKLIVG----------GMMG---SSEVGVELPFQLMHPKP-DA--
SAG_HUMAN LTLLPLLANNRERRGIALDGKIKHEDTNLASSTIIKEGIDRTVLGILVSYQIKVKLTVS----------GFLGELTSSEVATEVPFRLMHPQPEDP--
Arr3 struct eee eeeeeeeeeeeeee eeeeeeeee
Interactions # #
TXNIP KSLRVQKIRPS-----ILGCNILRVEYSLLIYVSVPGSKKVILDLPLVIG--SRSGLSSR---------TSSMASRTSSEMSWVDLNIPDTPEAPPCYMD
Phyre consensus eeecccccccc-----ccccceeeeeeeeeeeeeccccceeeeeeeeeee--cccccccc---------chhhhhcccccceeeeccccccccccchcc
Consens probab 77747899998-----688857999999999997599974788866998 5--46755544---------3566653445434553789889988966466
Arrestin domain ccccccccccccccccccccccccccccccccccccccccccccccccccccccccccccccccccccccccccccccccctttttttttttt

```
NP_001004605_ZF   IIT-DEERQCSSDLPAVRDEIE----------------------------GPLYA-------YIHEFRFQPP-------PLYSEVDPHPDYVCGSMDRRP
   ARRDC3_HUMAN   VVT-EEQRRNNLAPVSACDDFE------------------------RALQGPLFA-------YIQEFRFLPP-------PLYSEIDPNPD---QSADDRP
   Q6TEM6_ZFISH   VVT-EEEAAQNSTAVQPEEDLS------------------------GLLQHSLIA-------YMQEFRSRPP-------PVYSEVDPNPQP--ITMRRRC
   ARRDC2_HUMAN   VVADTEEAALGQSPFPLPQDPD------------------------MSLEGPFFA-------YIQEFRYRPP-------PLYSEEDPNPPL--GDMRPRC
   ARRDC4_HUMAN   VVP-EEEFSRHIPPYPQPPNCE------------------------GEVCCPVFA-------CIQEFRFQPP-------PLYSEVDPHPS---DVEESQP
XP_683827_ZFISH   ----------------------------------------------------------------------------------------------------
   Txnip_ZFISH    IT---RDCCIDQPLTPLLDDYD------------------------GG-DSPIFM-------NAAQFQFPPL-------PAYSEVEEEFNA----NARML
    TXNIP_HUMAN   VIP--EDHRLESPTTPLLDDMD------------------------GSQDSPIFM-------YAPEFKFMPP-------PTYTEVDPCILN----NNVQ-
NP_001017785_ZF   ----------------------------------------------------------------------------------------------------
NP_001017889_ZF   PPGQFSAPGNYGPPVGQFPSPGYPGPPLGQGGPPSYTGPPLGQFGAPAFPGPPGQ-------SAPSYYSGAPGQFGSSVPLQSSTSAPPPYQEYKFYPQL
XP_695840_ZFISH   PGG--AAP----PPAGLYPNLYDPVPP---------------------YPGTAAG-------TFPNPNAMLP-------GFHPAPSAPVYDQNQSTKESF
   Q6ZM71_ZFISH   PRPRSTHASPSAPPVDLYPQLPSVSNYNG-----------------EMLKSPHQEPGSQGPVSPNAFSYAPGLSFRQRQSSSGPSAPPPSLSSSSQDRNQ
   ARRDC1_HUMAN   PVFLSTKSHSQRQ-----PLLATLSSVPG-----------------APEPCPQDG-------SPASHPLHPP-------LCISTGATVPYFAEGSGGPVP
   ARRDC5_HUMAN   TRYELVTTVHLPW-----SLTSLKAKVP----------------------------------IIITSASVDS-------AICQLSEDGVLPVNPDHQN--
NP_999846_ZFISH   LSHSTSAVPMLDPPIDTNLIEF----------------------------------------DTNSLIPDDD-------IVFEDFARLRLKGVIDKE-ED
    Arrb2_ZFISH   NSRPQSAVPETDVPVDANLIEF----------------------------------------ETNNFSQDDD-------FVFEDFARLRLKGMKDEE-DD
    ARRB2_HUMAN   LPRPQSAAPETDVPVDTNLIEF----------------------------------------DTN-YATDDD-------IVFEDFARLRLKGMKDDDYDD
    Arrb1_ZFISH   --SIYRDAPENDAPIDTNLIEF----------------------------------------DTN----DDD-------IIFEDFARQRLIGAK------
    ARRB1_HUMAN   --PPHREVPENETPVDTNLIEL----------------------------------------DTN----DDD-------IVFEDFARQRLKGMKDDKEEE
AAX69083_ZFISH    ----------------------------------------------------------------------------------------------------
NP_001002405_ZF   ----------------------------------------------------------------------------------------------------
     Arr3_ZFISH   ------------------------------------------------------------------SDINLE----------------------------
     ARR3_HUMAN   ------------------------------------------------------------------AASSED-------IVIEEFTRKGEEESQKAVEAE
Zgc-66109_ZFISH   ---------------------------------------------------------------VKESEAEEE-------MVFEEFKRTYLKGMPEDEDEG
      SAG_HUMAN   ---------------------------------------------------------------AKESYQDAN-------LVFEEFARHNLKDAGEAEEGK

    Arr3 struct                                                                                  eeeee
   interactions                                                                                  ***    #

          TXNIP   VIP—EDHRLESPTTPLLDDMD------------------------GSQDSPIFM-------YAPEFKFMPP-------PTYTEVDPCILN----NNVQ
Phyre consensus   ccc—chhhccccccccccccc------------------------cccccchhe-------eehhhccccc-------cccccccchehc----cccc
  consens probab  788—5565689999988887------------------------667898454-------5355578989-------987656863427----8889

Arrestin domain   tttttttttttttttttttttttttttttttttttttttttttttttttttttttttttttttttttttttttttttttttttttttttttttttttttt
```

NP_001004605_ZF	DNCPSR---------------------------------
**ARRDC3_HUMAN**	SCPSR----------------------------------
Q6TEM6_ZFISH	MTC------------------------------------
**ARRDC2_HUMAN**	MTC------------------------------------
**ARRDC4_HUMAN**	VSFIL----------------------------------
XP_683827_ZFISH	---------------------------------------
Txnip_ZFISH	PVC------------------------------------
**TXNIP_HUMAN**	---------------------------------------
NP_001017785_ZF	---------------------------------------
NP_001017889_ZF	PDQPEKS--------------------------------
XP_695840_ZFISH	PNAPY----------------------------------
Q6ZM71_ZFISH	MPSSASVPPSYRSSAYPQEAPPSYEDSFNT---------
**ARRDC1_HUMAN**	TTSTLILPPEYSSWGYPYEAPPSYEQSCGGVEPSLTPES
**ARRDC5_HUMAN**	---------------------------------------
NP_999846_ZFISH	C--------------------------------------
Arrb2_ZFISH	HFC------------------------------------
**ARRB2_HUMAN**	QLC------------------------------------
Arrb1_ZFISH	---------------------------------------
**ARRB1_HUMAN**	EDGTGSPQLNNR---------------------------
AAX69083_ZFISH	---------------------------------------
NP_001002405_ZF	---------------------------------------
Arr3_ZFISH	---------------------------------------
**ARR3_HUMAN**	GDEGS----------------------------------
Zgc-66109_ZFISH	NANIEENV-------------------------------
**SAG_HUMAN**	RDKNDADE-------------------------------
Arr3 struct interactions	
TXNIP	
Phyre consensus	
consens probab	
Arrestin domain	

Additional file 4. Human arrestin family compared to select fish arrestins. We include all the high confidence zebrafish arrestins that we could identify, but there may be more [see Additional file 2]. Positions that may be conserved in both classes of vertebrate arrestins are shaded yellow, those specific to alphas are blue, beta are red, potentially notable are light gray, and PY motifs are black. A candidate class I SH3-binding motif (RXXPXXP) conserved in a loop near the center of the C domain of ARRDC2/3/4 is shown by dark gray shading of white letters. The arrestin domains are given at the bottom, n for N domain, c for C and t for Tail; italics show sequence not considered as part of the N and C domains according to Pfam. Shading on that line maps secondary structure elements on ARR3 (cone arrestin) – beta strands in gray and the one alpha helix in black (adapted from Sutton et al. 2005). Below the ARR3 structural elements, the predicted secondary structure of TXNIP is shown [see Additional file 2]. Underlining highlights regions involved in receptor specificity. Asterisks (*) show residues involved in the "three-element interaction"; number symbols (#) mark residues that make up the "polar core".
