# Supplementary material for: On the origins of arrestin and rhodopsin
Source: BMC Evol Biol. 2008 Jul 29;8:222. doi: 10.1186/1471-2148-8-222 (PMC2515105; doi:10.1186/1471-2148-8-222)
Supplement: Additional file 5 — Phylogeny of canarypox virus alpha arrestin. A PDF file showing the phylogeny of canarypox virus (CNPV) alpha arrestin. This analysis shows CNPV arrestin is a highly divergent vertebrate Arrdc3 that was acquired by horizontal gene transfer. [file 1471-2148-8-222-S5.pdf]

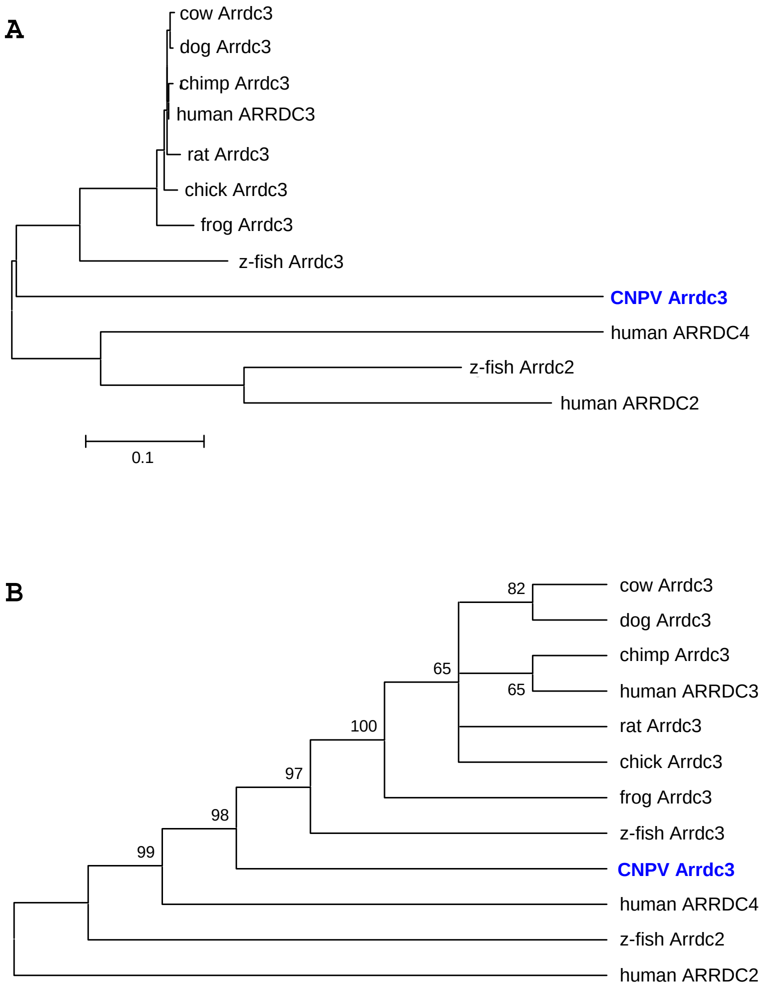
A
cow Arrdc3
dog Arrdc3
chimp Arrdc3
human ARRDC3
rat Arrdc3
chick Arrdc3
frog Arrdc3
z-fish Arrdc3
CNPV Arrdc3
human ARRDC4
z-fish Arrdc2
human ARRDC2
0.1
B
82
65
65
100
97
98
99
cow Arrdc3
dog Arrdc3
chimp Arrdc3
human ARRDC3
rat Arrdc3
chick Arrdc3
frog Arrdc3
z-fish Arrdc3
CNPV Arrdc3
human ARRDC4
z-fish Arrdc2
human ARRDC2

Additional file 5. Canarypox virus (CNPV) alpha arrestin phylogeny. We created a neighbor-joining tree (amino acid pairwise distance, with Poisson correction) of the GenBank sequences most closely related to CNPV arrestin, and show two representations. The same tree was obtained by Maximum parsimony tree building. (A) Neighbor-joining tree with branch lengths corresponding to the number of changes per amino acid position (scale bar). (B) Consensus bootstrap tree from 500 repetitions. CNPV is highly divergent, but it is clearly an Arrdc3 orthologue.
