# Supplementary material for: On the origins of arrestin and rhodopsin
Source: BMC Evol Biol. 2008 Jul 29;8:222. doi: 10.1186/1471-2148-8-222 (PMC2515105; doi:10.1186/1471-2148-8-222)
Supplement: Additional file 6 — Helix I region in vertebrate arrestins. A PDF file showing evidence that helix I of visual/beta arrestins is absent in alpha arrestins. Protein sequence alignment and secondary structure prediction of alpha arrestins suggests they lack the helix I present in beta arrestins. [file 1471-2148-8-222-S6.pdf]

```
arrestin domain      nnnnnnnnnnnnnnnnnnnnnnnnnnnnnnnnnnnnnnnnnnnnn

      fish Arr3      FEGTKP--PNTPMQEALLKKAG-DQGHPFTFDIPVHLPCSVSLQP
    fish Q7ZZA6      FEGTKP--PNTPMQEALLKKAG-DQGHPFTFDIPVHLPCSVSLQP
 fish Zgc-92697      EAGHKP--TLTEMHNTLLKKAG-EQGHPFTFNIPTNLPCSVTLQP
     human ARR3      AESSSPQGALTVLQERLLHKLG-DNAYPFTLQMVTNLPCSVTLQP
 fish Zgc-66353      PIPEESK-PHSRLQERLLKKLG-QNAYPFHFSIPQNLPCSVTLQP
    human ARRB2      PVPNPPR-PPTRLQDRLLRKLG-QHAHPFFFTIPQNLPCSVTLQP
    human ARRB1      PAPEDKK-PLTRLQERLIKKLG-EHAYPFTFEIPPNLPCSVTLQP
 fish Zgc-66109      ALQDKEQCILTKVQEKLLRKLG-DNAYPFFFEFPDNLPCSVGLQP
      human SAG      PVG--AASTPTKLQESLLKKLG-SNTYPFLLTFPDYLPCSVMLQP
     fish Txnip      DEHPTDN-----DGSVIL-RPGNKYEYSFGFELPAQGQL-VSSYK
    human TXNIP      EDQPTGE-----NEMVIM-RPGNKYEYKFGFELP-QGPL-GTSFK
 fish Zgc-85652      AD--NGD-------LTVI-PAG-RHEYPFSFQLP-EETL-VTSFE
   human ARRDC2      PD--TGE-------TTTL-PPG-RHEFLFSFQLP--PTL-VTSFE
   human ARRDC3      HERDDDN---SEEGFHTI-HSG-RHEYAFSFELP-QTPL-ATSFE
   human ARRDC4      PP--AGE------GIILL-QPG-KHEFPFRFQLP-SEPL-VTSFT
    fish Q6ZM71      KG---------------TL-KEG-EHSFPFKFLLP--AAA-PTSFV
   human ARRDC1      KG---------------SL-PAG-EHSFPFQFLLP--ATA-PTSFE
   human ARRDC5      ED--------------NWL-SAG-SHTFDFHFNLP--PRL-PSTFT

ARRB1 2ry struc.     PAPEDKK-PLTRLQERLIKKLG-EHAYPFTFEIPPNLPCSVTLQP
            BPS      CCCCCCC-HHHHHHHHHHCCCC-CCCEEEECCCCCCCCCCCCCCC
            D_R      CCCCCCC-CCCHHHHHHHHCHC-CCHCCCCCCCCCCCCCCECCCC
            DSC      CCCCCCC-CHHHHHHHHHHHHC-CCCCCEEEECCCCCCCEEEECC
            GGR      CCCCCCC-CHHHHHHHHHHHCC-CCCCCCEEEECCCCCCCEEECC
            GOR      CCCCCCC-HHHHHHHHHHHHHH-CCCCCCECCCCCCCCCCCEECC
            H_K      CCCCCCC-CCHHHHHHHHHHHH-CCCCCCCCCCCCCCCCCCEECC
            K_S      CCCCCCC-CCCCHHHHHHHHHH-HCCCCCCCCCCCCCCCCCCCCC
            JOI      CCCCCCC-CHHHHHHHHHHHHC-CCCCCCECCCCCCCCCCCEECC

ARRDC2 predict.      PD--TGE-------TTTL-PPG-RHEFLFSFQLP--PTL-VTSFE
            BPS      CC--CCC-------CCCC-CCC-CCCCCCCCCCE--EEE-EEEEC
            D_R      CC--CCC-------CCCC-CCC-CHHHHEEECCC--CCE-EEEEC
            DSC      CC--CCC-------EEEC-CCC-CEEEEEEECCC--CCE-EEEEC
            GGR      CC--CCC-------CEEC-CCC-CCCEEEECCCC--CCE-EEECC
            GOR      CC--CCC-------EEEC-CCC-CHHEEHHHCCC--CCE-EEEEE
            H_K      CC--CCC-------EEEC-CCC-CCCEEEECCCC--CCE-EEEEE
            K_S      CC--CCC-------CCCC-CCC-CCCEECCCCCC--CCE-ECCCC
            JOI      CC--CCC-------CEEC-CCC-CCCEEEECCCC--CCE-EEEEC
```

Additional file 6. Visual/beta arrestin helix I is absent in alpha arrestins. The multiple sequence alignment shows visual/beta arrestins (red/top) and alpha arrestins (blue/bottom) from zebrafish and humans. Positions that may be conserved in both vertebrate subfamilies are shaded yellow, those specific to alphas are blue, visual/betas are red, and potentially notable are light gray. Below, is the ARRB1 sequence duplicated from the alignment, but with the secondary structure features determined by crystallography (adapted from Han et al. 2001) mapped – beta strand in gray and helix in black. Below that are the ARRB1 secondary structure predictions made by eight algorithms (PELE, UCSD Biology Workbench [see Additional file 2]. The helix is predicted to be absent in all human alpha arrestins. Below that is the ARRDC2 sequence duplicated, shown with its secondary structure predictions. The top line shows the aligned region is within the Arrestin N domain.
